# The gut-microbiota-endometriosis axis: a review of immune regulation, microbial dysbiosis, host transcriptomics, probiotic intervention, and dietary influences

**DOI:** 10.3389/fimmu.2026.1947426

**Published:** 2026-10-19

**Authors:** Vyshnav S, Sajitha Lulu S

**Affiliations:** School of Bio Sciences and Technology, Vellore Institute of Technology (VIT), Vellore, India

**Keywords:** Endometriosis, gut microbiota, immuno-inflammatory networks, probiotics, transcriptomics, dietary determinants

## Abstract

Endometriosis is a persistent gynaecological disease state marked by development of endometrial-like tissue exterior to the uterus, which triggers pelvic discomfort, chronic inflammation, and fertility issues. Although it has been associated with hormonal imbalances and immune dysfunction, emerging research describes a major role for the microbiota in initiating and accelerating the disease. Disruptions in the gut microbiota have been associated with immune system activation and inflammatory signaling, which may intensify development of endometrial lesions. This review delves into recent findings on how microbial imbalances contribute to the inflammatory landscape of endometriosis, emphasizing the involvement of specific microbial species in influencing estrogen pathways and immune regulation. Therapies centred on probiotics especially strains like *Lactobacillus* and *Bifidobacterium* are gaining attention for their potential to rebalance the microbiota, strengthen epithelial barriers and mitigate inflammation. Also, the gene expression studies have shed light on molecular mechanisms affected by microbial alterations, revealing new biomarkers and therapeutic targets. These insights highlight the growing relevance of microbiome research in deepening our understanding of endometriosis and refining its treatment. This review is novel in its comprehensive integration of microbiome, immunological, and molecular insights into endometriosis, and it highlights the potential of microbiota-based diagnostics and individualized probiotic strategies as innovative clinical interventions beyond conventional hormone-centred approaches.

## Graphical Abstract

**Figure:**
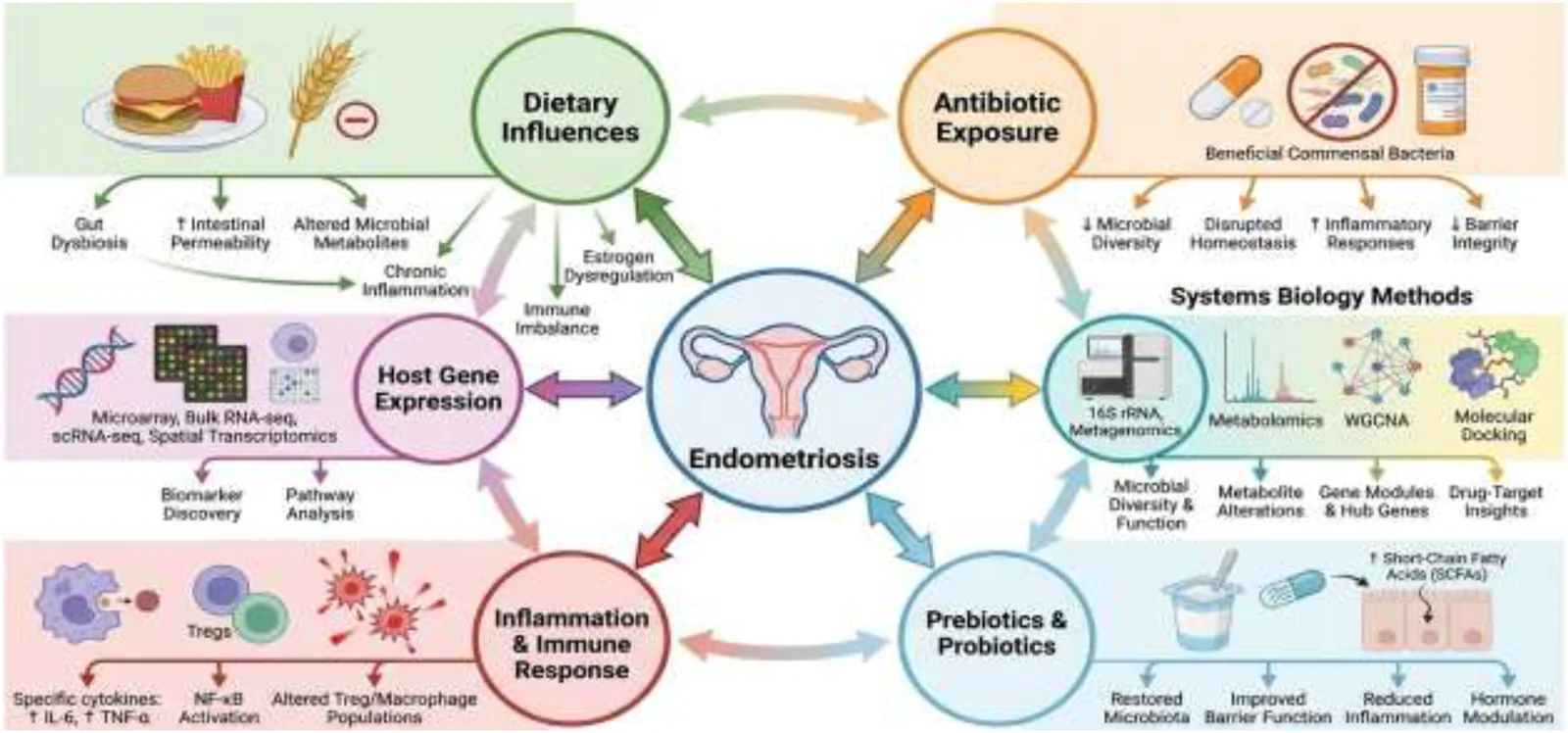

## 1 Introduction

Endometriosis is identified as a chronic inflammatory disorder influenced by estrogen, characterized by the development of endometrial tissue beyond the uterus, primarily impacting women of reproductive stage. It initiates a long-term inflammatory response that can result in the expansion of scar tissue, such as adhesions and fibrosis particularly within the pelvic cavity, potentially affecting the reproductive organs, bowel, urinary tract, and surrounding tissues. in the pelvic region and other parts of the human body (1). Endometriosis affects approximately 6-10% of women of reproductive stage (2). As illustrated in **Figure 1**, the prevalence of endometriosis has steadily increased over the past few decades. Although the endometriosis is highly predominant and greatly affect the quality of life, its underlying causes are still not fully understood (3).

**Figure 1:**
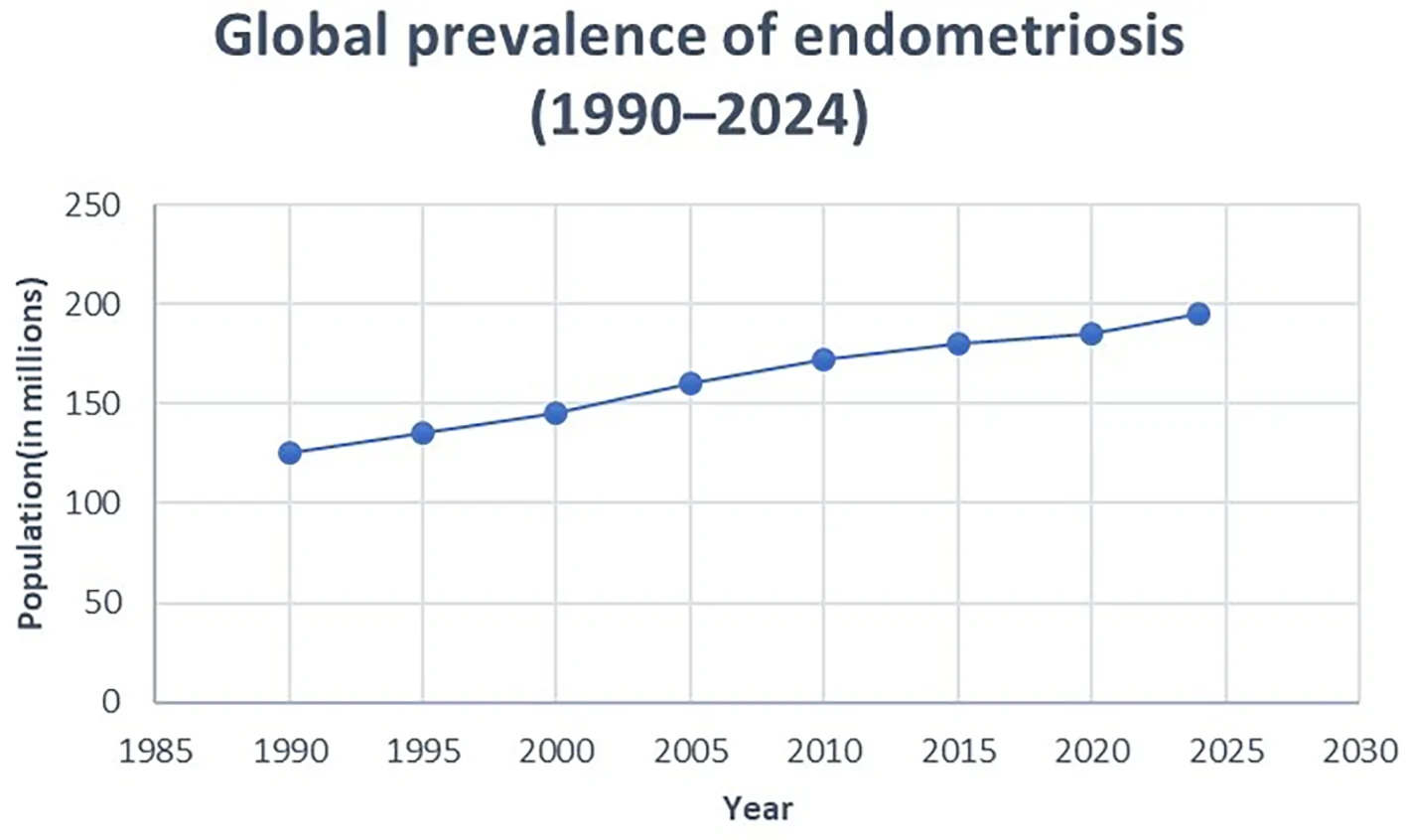
Global prevalence trends of endometriosis (1990–2024). This figure depicts the changes in the global prevalence of endometriosis over study period, demonstrating a progressive increase in disease burden worldwide. This shows the need, awareness, early diagnosis, and the development of effective preventive and therapeutic targets.

The female reproductive tract (FRT) consist of discrete microbial communities at different regions. The vagina is usually dominated with the *Lactobacillus* community and has low microbial diversity, whereas the upper tract which covering uterus, endocervix, fallopian tubes, and ovaries hosts various bacterial populations though the composition, persistence, and biological significance of these microbial communities remain under investigation (4). A healthy FRT relies on a precisely adjusted interaction between the immune system and resident microbiota, which results sustainable immune tolerance (5). Any disruption in this equilibrium which is caused by a shift in microbial composition, immune alterations or environmental factors can lead to dysbiosis. In endometriosis, studies report a higher abundance of bacterial groups such as Proteobacteria, Bacillota, Enterobacteria, Corynebacteria and Pseudomonadota along with notable reduction in *Lactobacillus* species that can correlate with increased inflammatory activity (6). Moreover, the detection of more than 10% of non-*Lactobacillus* species in the uterine microbiota will cause implantation failure and pregnancy loss which reveals that microbial influences the healthy vaginal environment (5). These findings provide relevant background on the potential role of microbial dysbiosis in reproductive health; however, they are presented here only as contextual information and fall outside the inclusion criteria and search strategy of the present review. The current review focuses specifically on the gut microbiota and its potential contribution to endometriosis through immune, inflammatory, metabolic, and gut-reproductive tract interactions. Beyond benign gynaecological conditions, alterations in the local reproductive-tract microbiota have also been implicated in gynaecological oncology, offering a useful comparative perspective for the dysbiotic patterns discussed throughout this review. In endometrial carcinoma, case-control and narrative-review evidence similarly describes a shift away from ma *Lactobacillus*-dominant profile toward greater microbial diversity and enrichment of pro-inflammatory, potentially genotoxic taxa, a pattern that parallels the *Lactobacillus* depletion and pro-inflammatory taxon enrichment reported in endometriosis (7, 8). Mechanistically, both conditions converge on chronic low-grade inflammation, NFKB-driven cytokine signaling, and dysregulated local estrogen metabolism as candidate drivers linking dysbiosis to disease, suggesting that the microbiota-inflammation-estrogen axis described throughout this review may reflect a shred, cross-cutting mechanism relevant to both benign and malignant gynaecological pathology rather than one unique to endometriosis. endometrial carcinoma and endometriosis differ substantially in cellular biology and clinical trajectory, and the current cross-condition microbiota evidence remains correlative and case-control in design; it is presented here for comparative context only and does not extend the inclusion criteria of the present review.

The higher occurrence and chronic nature of the endometriosis will cause reduced quality of life persist despite current treatments. Around 33% of patients are weak responders to the progestins, oral contraceptives and the progesterone resistance limits the efficacy of hormone-based treatment (9). In certain cases, it has been observed that surgical removal of endometriotic lesions results in recurrence in up to 50% of patients within five years. Thus, surgery alone is not sufficient to halt disease progression or prevent its return (10). Treatments involving GnRH agonists and antagonists often lead to hypoestrogenic complications such as hot flashes, reduced bone mineral density, and mood disturbances, making long-term use impractical for many patients. Due to the complex and multifactorial nature of endometriosis which involves inflammation, oxidative stress, immune imbalance, and microbiota disruption emerging non hormonal therapies targeting immune pathways, oxidative stress, and regulatory RNAs are being explored as promising alternatives (11).

The gut-reproductive axis mutual communication network through which gut microbiota influences reproductive physiology, hormonal balance, immune responses and inflammatory pathways. Upcoming evidences suggest that microbial metabolites, endocrine signals and immune mediators act as a messenger enabling the cross-talk, thereby connecting intestinal health stability with reproductive tissue function. Disruptions in the gut microbiota have been associated with abnormal estrogen processing, weakened immune tolerance, and persistent inflammation are the key mechanisms now understood to contribute to disorders like endometriosis, infertility, and polycystic ovary syndrome (PCOS) (12). Moreover, the enterohepatic recycling of estrogen controlled in part by microbial beta-glucuronidase enzyme demonstrates how gut bacteria can shape circulating hormone levels and effects the reproductive tracts local environment (13). The estrobolome comprises the collective supply of gut microbial genes and metabolic activities involved in estrogen metabolism, particularly β-glucuronidase (GUS) and GUS-like enzymes found in bacterial taxa such as *Clostridia, Bacteroides, Escherichia, and Lactobacillus.* These enzymes can deconjugate heaptically glucuronidated estrogens in the intestinal lumen, thereby facilitating their reabsorption through the enterohepatic circulation and reducing their elimination in feaces (10, 14). Alterations in the composition and functional activity of the gut microbiota, including enrichment of GUS- producing bacteria, may consequently influence the availability of biologically active unconjugated estrogens and potentially contribute to the estrogen- responsive environment associated with endometriosis. These kind of association have been reported to exhibit differences in faecal and vaginal β- glucuronidase activity compared with disease-free controls, alongside alterations in microbial composition, including reduced abundance of *Lactobacillus* (15). Although these observations suggest that the estrobolome may represent an important interface between gut microbial dysbioisis and estrogen- dependent endometriosis, the extent to which altered microbial estrogen metabolism contributes directly to lesion development and progression remain to be established. The potential pathway also intersect imuune-inflammatory signaling, antibiotic-induced microbial perturbation, dietary influences and microbiome- targeted therapeutic approaches, highlighting the broader relevance of the estrobolome to the pathophysiology and clinical management of endometriosis. Collectively this evidence emphasizes that microbial cross talk is fundamental to sustaining reproductive inflammation and disrupted estrogen signaling associated with endometriosis can modify gut microbial composition, establishing a self-reinforcing cycle that promotes disease persistence. Studies indicate that individuals with endometriosis frequently display gut dysbiosis accompanied by elevated pro-inflammatory cytokines and reduced immune tolerance.

This review investigates the evolving link between microbiota and endometriosis, illustrating the role of microbial imbalance in driving inflammation and immune modulation, altering host gene expression, and shaping potential new treatment strategies. By integrating insights from microbiome research, transcriptomics, and immunology, it aims to provide a comprehensive understanding of the dynamic interactions between microbial populations and the immune system in the context of endometriosis (16). Importantly, this work distinguishes itself by consolidating emerging evidence on the interplay between microbiota composition, immune dysregulation, and disease pathophysiology, an area that remains inadequately addressed in existing literature. By bridging microbiome research with immunological and molecular perspectives, it offers a novel framework to explain how microbial dysbiosis not only perpetuates chronic inflammation but also drives disease progression and therapeutic resistance. This integrative perspective moves beyond conventional hormone-centric approaches, opening avenues for microbiota-targeted and immune-modulatory therapies. From both scientific and societal standpoints, such insights hold the potential to redefine paradigms in women’s reproductive health research, contributing to the progression of innovative non-hormonal interventions that may enhance clinical outcomes and alleviate the global burden of endometriosis. Although upcoming evidence implicates that the microbiota plays a major role in the pathophysiology of endometriosis, the available literature remains fragmented. Previous reviews have widely focused on individual aspects, such as microbial dysbiosis, immune dysregulation, probiotic interventions, dietary factors or host transcriptomic alterations. Rather than integrating these interconnected components into a comprehensive framework. To date, the available reviews have not integrated these interrelated domains into a comprehensive framework that explains the complex mechanisms driving the onset and the endometriosis progression. To address and fill this gap, the present review introduces the integrated gut microbiota-endometriosis axis, a conceptual model that connects microbial dysbiosis with immune activation, chronic inflammation, microbiota-mediated alteration in estrogen metabolism, and host transcriptomic changes. By incorporating these interconnected biological processes, this framework provides a holistic understanding of how they collectively promote lesion development, pain, infertility, disease progression, and recurrence while highlighting potential therapeutic targets through microbiota modulation. By combining the antimicrobial perturbations, probiotic modulation, and dietary determinants in this framework which is important to determine the environmental factors that influence gut microbial composition and modulate interconnected molecular, endocrine and immune pathways. Such an integrated approach is crucial because endometriosis is a multifactorial disease arising from complex interaction among the gut microbiota, immune responses, hormonal regulation, host transcriptomic networks, and environmental impacts. Evaluating these components in isolation provides only a partial understanding of the biological processes underlying disease heterogeneity, progression, and variability in therapeutic outcomes. By adopting a systems-level perspective, this review offers a comprehensive framework for understanding host-microbiota interaction in endometriosis, identifies promising biomarkers and therapeutic targets and provides a foundation for the development of microbiota- based diagnostics, personalized probiotic therapies, and personalized medicine approaches.

## 2 Methods

The study selection for this review was carried out based on the PRISMA 2020 guidelines, ensuring methodological reproducibility and transparency. The objective of the review was to comprehensively synthesise the proof on the involvement of the gut microbiota in endometriosis by incorporating findings on immune-inflammatory pathways, antimicrobial perturbations, probiotic modulation, host-transcriptomic alterations, and dietary determinants. The review was conducted in accordance with the PRISMA 2020 guidelines and compromised identified the literatures, screened the study, carried eligibility analysis, methodological quality appraisal and qualitative data synthesis to ensure a transparent and reproducible review process. A comprehensive literature search was conducted across PubMed, Scopus, and ScienceDirect to identify relevant studies. The literature search was conducted between 15 and 31 March 2026 and covered all articles published up to 31 March 2026. The search strategy used combinations of Medical Subject Headings (MeSH), indexed terms, and free text keywords related to endometriosis, gut microbiota, inflammation, immune responses, transcriptomics, probiotics, antimicrobial exposure, and dietary factors. Boolean operators (OR and AND) were employed to integrate search terms, and the search strategy was tailored for each database to accommodate differences in indexing schemes and search capabilities. To maximise study identification, the reference lists of all included and relevant review articles were manually examined to identify additional eligible publications that were not captured by electronic database searches. Eligible records were original research articles reporting empirical data on gut microbiota, host transcriptomic signatures, dietary intake, or probiotic intervention in relation to endometriosis. Systematic reviews and meta-analyses identified during the search were not included in the qualitative synthesis but were screened manually to identify additional primary studies meeting the eligibility criteria.

Studies were included if they fulfilled the following eligibility criteria: (1)involved human participants with a confirmed diagnosis of endometriosis and relevant animal models investigating the association between endometriosis; (2)evaluated gut microbiota composition, microbial dysbiosis, or host-microbiota interactions; (3)investigated immuno-inflammatory mechanisms, antimicrobial exposure, probiotic interventions, host transcriptomic alterations, or dietary factors associated with endometriosis; (4)were original research articles or systematic reviews published in peer- reviewed scientific journals were manually screened, including their reference lists; (5)provided full-text access; and (6)were only published in English. Also studies were excluded if they fulfilled the following eligibility criteria;(1)did not focus on endometriosis; (2)failed to recognize the microbiome-related mechanisms and investigate gut microbiota; (3)were letters to the editors, conference abstracts, editorials, case reports, book chapters or other non-peer –reviewed publications;(4)lack of relevant methodological data details or relevant outcome data;(5)represented duplicate records;(6)were only published in languages other than English.

We performed a comprehensive search of the PubMed database employing the Boolean query (“Endometriosis” OR “EM”) AND (“gut microbiota” OR microbiota OR microbiome OR dysbiosis OR “gut bacteria”) AND (inflammation OR immunity OR immunology OR transcriptomics OR RNA-seq OR probiotics OR probiotic OR diet OR dietary OR nutrition OR antimicrobial OR antibiotics) with additional searches conducted using related terms all-embracing inflammation, transcriptomics, probiotic and dietary factors to retrieve studies investigating the association between endometriosis and the gut microbiome. In the identification stage, a total of 3047 records were obtained from major electronic databases, including PubMed (1061), Scopus (760), and ScienceDirect (1226). Following the removal of 614 duplicate records and the exclusion of 218 entries through automated screening tools and 165 records in other criteria such as incomplete records or retracted records etc., 2050 unique records were retained for further assessment. During screening phase, the titles and abstracts of these 2050 records were evaluated for relevance to association between gut microbiota and endometriosis. A total of 1612 records were excluded due to irrelevance, resulting in 376 articles for eligibility evaluation. In eligibility phase, full-text articles were thoroughly evaluated based on predefined inclusion and exclusion criteria of these 296 studies were excluded due to reasons such as absence of microbiome- related focus, absence of relevance to endometriosis, insufficient data, or classification as non-original research. Ultimately, in inclusion phase, 80 studies satisfied all eligibility requirements and were included in the qualitative synthesis. The PRISMA flow diagram illustrating the study selection process is presented in **Figure 2**. These selected studies provided comprehensive insights into the complex interaction between gut microbiota and endometriosis, encompassing aspects such as inflammatory mechanisms, immune regulation, antimicrobial effects, probiotic interventions, host transcriptomic responses and dietary influences.

**Figure 2:**
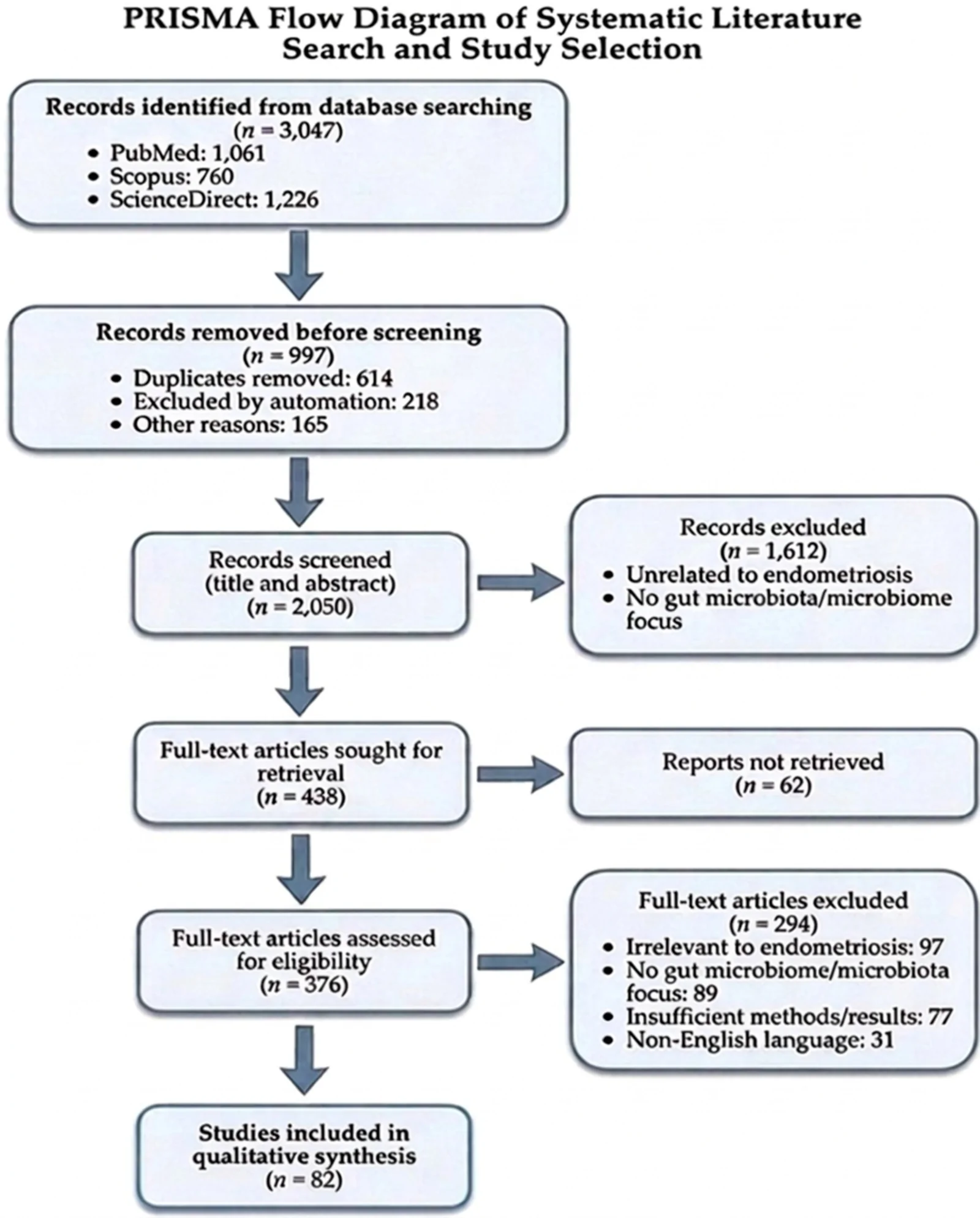
PRISMA flow diagram of study selection. The flow diagram illustrates the identification, screening, eligibility assessment and final conclusion of studies following the PRISMA 2020 guidelines. records are retrieved from multiple electronic databases were screened after duplicate removal, followed by full-text eligibility assessment based on predefined inclusion and exclusion criteria, resulting in the final studies included in the review.

## 3 Inflammation and immune modulation

The intestine is a significant immune organ where a wide population of immune cells is present. The microbiota in the gut is participated in the regulation of innate as well as adaptive immune responses that maintains the intestinal homeostasis. The pathogen associated microbial patterns like lipopolysaccharide (LPS), flagellin, peptidoglycan, short-chain fatty acids and bile acids are the key players in the regulation of the host-microbiota interaction (17). These molecules interact with the pattern recognition receptors, especially Toll-like receptors expressed on the epithelial cells in the intestine which results in the release of inflammatory cytokines, chemokines, and growth factors like IL-1, IL-3, IL-6, and TNF-alpha, which regulate immune response simultaneously (17).

In terms of cytokines, endometriosis is marked by a pro-inflammatory environment with upregulated IL-6, TNF-alpha, and IL-17 coupled with NF-kB activation in both peritoneal fluid and endometrial lesions. All these factors facilitate endometriosis cell survival, adhesion, and angiogenesis. All these cytokines have been found in patient cohorts with endometriosis. Moreover, these cytokines have been reproduced in endometriosis models where estradiol and microbial ligands cooperate to enhance macrophage IL-6/TNF secretion via NF-kB activation (18).Compositional changes in immune cells represent another hallmark of endometriosis. Specifically, Treg/Th17 imbalance with reduced FOXP3+ Tregs and relatively enhanced Th17 responses, as well as dynamic macrophage polarization, have been identified in endometriosis. Early-stage endometriosis lesions contain M1 macrophages with a pro-inflammatory profile, whereas advanced endometriosis is marked by increased by M2-like macrophages with a pro-tissue modeling profile. The latter is possibly mediated by microbial ligands in a local microenvironment (19).

Histamine-mediated immune signaling contributes to this inflammatory landscape, as indicated by the upregulation of histidine decarboxylase (HDC) and elevated systemic histamine concentrations. The widespread expression of histamine receptors (HRH1-HRH4) on immune cell, epithelial cells and nerve fibres supports a neuro-immune interface that promotes cytokine release, nerve growth factor activation, and chronic pain sensitization (17). Another mechanism contributing to the pathogenesis is innate immune dysregulation which is marked by the M2 macrophage polarization phenotype, which facilitates tissue healing, angiogenesis, and immune tolerance instead of ectopic cell elimination, along with decreased natural killer cell cytotoxicity which allows for immune escape; moreover, the complement system activation, especially C3a and C5a molecules, increases inflammation and angiogenesis. Adaptive immune alterations are also evident, characterised by a shift towards a Th2-dominant response and increased regulatory T cells, which collectively suppress immune surveillance and facilitates lesion persistence alongside B-cell activation and autoantibody production that sustain chronic inflammation. It should be highlighted that the endometriosis microenvironment displays a peculiar cytokine profile association with the simultaneous presence of pro-inflammatory and immunosuppressive cytokines such as IL-10 and TGF-beta (20).On a molecular level, several crucial pathways include PI3K/AKT and Wnt/β-catenin which control cellular proliferation, survival, and immune escape, as well as act as activation of COX-2, MMP-9, and IDO1 pathways leading to extracellular matrix remodeling, invasion, and immune suppression, especially in deep infiltrating lesions (21). Moreover, the interaction between immune system and nervous tissue is one of the characteristics of endometriosis-related pain associated with increased lesion intervention and immune cell/nerve fibre crosstalk via histamine, nerve growth factor and prostaglandin E2 causing sensitization of nociceptors and resulting in chronic pelvic pain (20). Finally, epigenetic alterations play a major role in immune-regulation in terms of increased methylation dependent repression of HOXA10 causing cytokine signaling alteration, impaired immune balance and loss of endometrial receptivity which promotes lesion persistence and infertility (22). Altogether, these data demonstrate the complexity of pathogenic mechanisms underlying endometriosis characterised by interaction of chronic inflammatory processes and immune disturbances which involves the estrogen–progesterone–autophagy pathway dysfunctional innate and adaptive immunity, and molecular pathways which favor lesion survival, angiogenesis, and chronic pain (17, 21). The major immune-inflammatory mediators, their regulatory patterns, functional implications, and potential associations with microbiota-driven mechanisms are summarized in **Table 1**. As depicted **Figure 3**, gut microbial dysbiosis represents an early event that influences multiple downstream pathological processes. It is characterised by reduced microbial diversity and increased abundance of pro-inflammatory microorganisms, contributing to impaired intestinal barrier function and elevated circulating LPS levels, leading to sustained activation of innate immune responses. The enhanced microbial beta- glucuronidase activity facilitates the de-conjugation of estrogen metabolites in the intestine. Altogether, the resulting chronic inflammatory microenvironment support the establishment, progression, and survival of endometriotic lesions.

**Table 1:** Differential regulation of immune and inflammatory pathways in endometriosis and their association with microbiota-driven mechanisms.

Gene / pathway	Regulation in endometriosis	Functional implication	Evidence / link to microbiota	References
IL6	Upregulated	Promotes inflammation, lesion proliferation, angiogenesis	Enhanced in peritoneal fluid and serum of patients; inducible by LPS/estradiol-TLR4 signaling.	(18, 57)
TNF (TNF-α)	Upregulated	Stimulates IL-6 release, NF-κB activation, adhesion/angiogenesis	Upregulated in lesions/peritoneal fluid; responsive to microbial ligands.	(57)
IL17 / Th17 pathway	Upregulated	Pro-inflammatory, promotes neutrophil recruitment and chronic inflammation	Increased Th17 responses reported in endometrium/peritoneum; linked to dysbiotic microbial signals	(64)
NF-κB signaling	Upregulated	Central transcriptional driver of inflammatory cytokines in stromal cells	NF-κB activation demonstrated in endometriotic stromal cells upon TNF/LPS stimulation	(18, 65)
FOXP3 (Treg marker)	Downregulated	Loss of immune regulation; higher Th17/Treg ratio	Reduced regulatory T cell signatures reported; implicated in implantation/lesion tolerance.	(54)
Macrophage polarization (M1→M2 shift with disease progression)	Stage-dependent (early M1, advanced M2)	Early inflammatory clearance vs later tissue remodeling/fibrosis	Flow cytometry and transcriptomic studies show stage-specific M1/M2 patterns; microbiota and LPS influence macrophage memory/polarization.	(19)
SIGIRR (negative regulator of TLR/IL-1R signaling)	Downregulated	Loss of negative feedback on inflammation	Decreased expression in ectopic tissue from transcriptomic analyses	(23)
TRAF4 / MAPK1 / DUSP4 (MAPK / TNF signaling components)	Downregulated	Altered MAPK/TNF signaling balance in lesions	Identified in immune-inflammation gene signatures from transcriptomic datasets	(23)
ACKR1, LMNB1, MFAP4, NMU, SEMA3C	Altered (diagnostic signatures)	Linked to macrophage activation and immune cell infiltration	Reported as part of macrophage/immune-related diagnostic gene set.	(66)

This table summarizes the direction of regulation, biological functions, microbiota-related evidence, and supporting references for major immune-inflammatory mediators involved in the pathogenesis of endometriosis.

**Figure 3:**
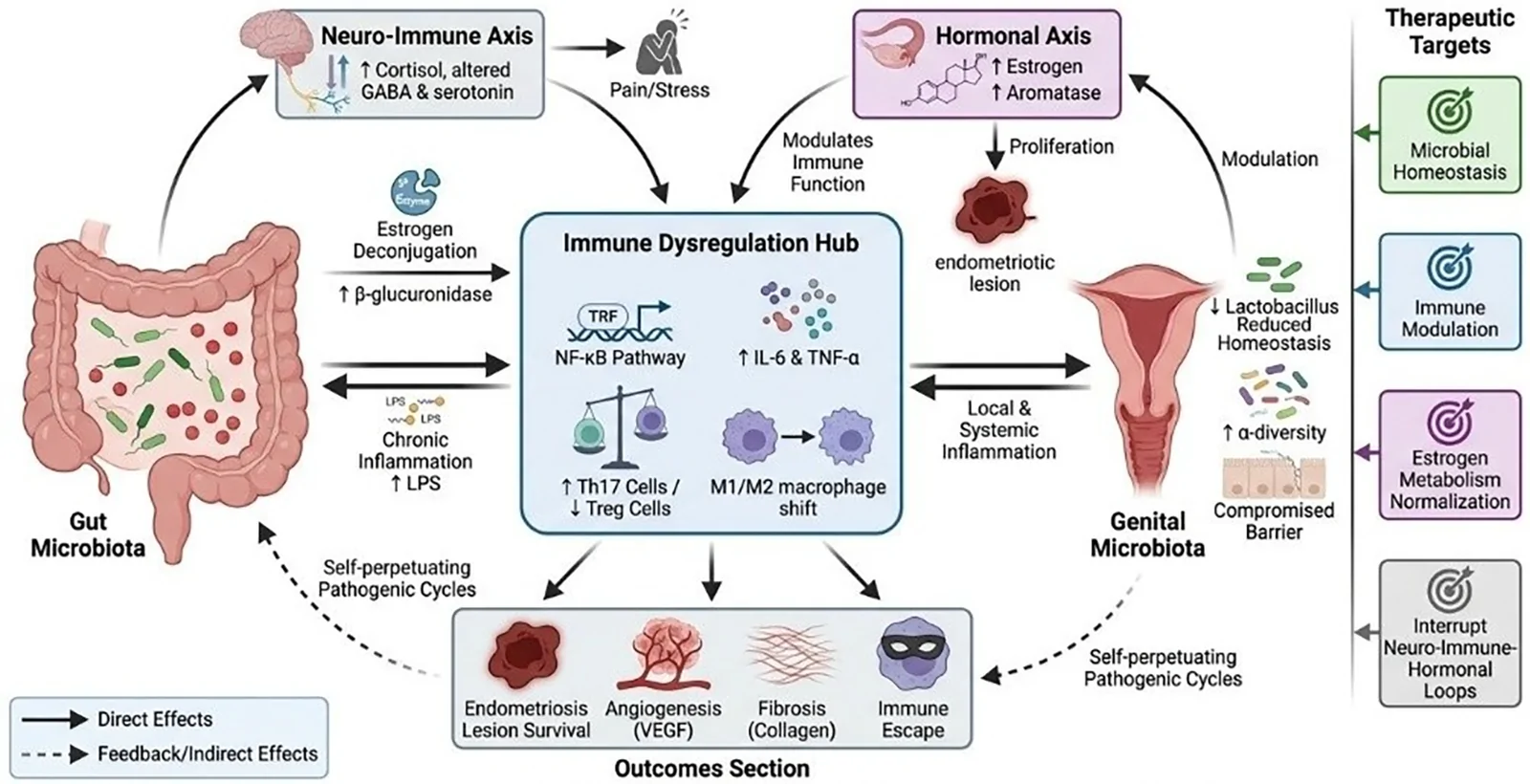
Gut–genital microbiota crosstalk in endometriosis. The schematic illustrates the bidirectional communication between the gut and genital microbiota and its contribution to the enhancement and progression of endometriosis. These interconnected microbial, immune, and endocrine interactions establish pathogenic feedback loops promote lesion survival, angiogenesis, fibrosis, evasion and chronic pelvic pain.

Endometriosis involves complicated immunological disorders not limited to the development of local inflammation but also associated with the alteration of immune genes’ expression and regulation systemically. Transcriptomic analysis suggests the presence of widespread dysregulation of immune/inflammation-related genes in endometriotic lesions; moreover, even the eutopic endometrium manifests considerable changes in the expression of such genes, pointing to the existence of innate immunological imbalance that can contribute to disease development (23). Besides, autophagy is a hormonally regulated cellular process that is closely associated with immune-cell dynamics, endometrial shedding, tissue repair, remodeling and cellular debris clearance during menstruation. In endometriosis. altered autophagic activity has been associated with dysregulated immune responses and may contribute to the persistence of ectopic endometrial tissue. Thus, the connection between hormonal imbalance and the dysfunction of immunity may involve in the estrogen-progesterone-autophagy pathway. Moreover, disrupted processes of autophagy and immune gene dysregulation interfere with normal mechanisms of clearing away debris, perpetuate inflammation, and further contributing to macrophage dysfunction and disruption of the cytokine signaling pathway (24). Summarizing, it should be noted that endometriosis is more than just a disease caused by local inflammation but represents a systemic disorder of immune system transcriptional reprogramming and malfunctioning.

Growing evidence indicates that gut microbiota dysbiosis plays a pivotal role in the chronic inflammatory environment and immune dysfunction underlying endometriosis. Disruption of the intestinal microbial ecosystem can diminish the gut barrier integrity, facilitating the translocation of bacterial lipopolysaccharide (LPS) into the bloodstream. Circulating LPS activates the toll-like receptor (TLR4) signaling cascade, thereby stimulating the production of pro-inflammatory mediators such as TNF-alpha, IL-1beta and IL-6. Nourished activation of these inflammatory pathways promotes macrophage infiltration, angiogenesis, survival of ectopic endometriosis lesions, and disease progression. In clinical trial, it was found that the women with endometriosis exhibited uplifted fecal inflammatory biomarkers, including beta-glucuronidase and secretory immunoglobulin A(slg A), providing additional evidence that gut dysbiosis contributes both systemic immune activation.

Although these evidence highlights the importance of gut-microbiota-immune axis in the development and progression of endometriosis, interpreting the available findings remains challenging because of the important methodological differences across studies and several potential source of bias. The current literature contains a wide range of study designs, such as cross-sectional, case-control, experimental studies and longitudinal, with considerable variation in sample size, patient characteristics, disease severity, and type of biological samples analyzed including feces, serum, eutopic and ectopic endometrial tissues and peritoneal fluids. The methodological inconsistencies make direct comparisons across studies difficult and likely explain the conflicting results regarding microbial compositions and immune alterations. Many clinical studies conducting relatively small patient cohorts and are based on cross sectional designs, limiting statistical power and preventing conclusions about causality. Other important limitation such as menstrual cycle phase, hormonal therapy, body mass index (BMI), dietary habits, antibiotic use, and lifestyle. Experimental animal models have provided valuable mechanistic evidence by demonstrating that depletion of gut microbiota can reduce lesion growth, macrophage infiltration, and inflammatory cytokine production. Many of these studies lack standardized validation, randomization and blinding. Despite the limitations, recent studies have strengthened the field by integrating microbiome, immunological, metabolomic, and transcriptomic analyses, providing deeper insights into gut microbiota-host interactions in endometriosis. Such approaches will also improve our understanding of how gut microbial dysbiosis drives chronic inflammation and immune regulation, also facilitating the identification of robust inflammatory biomarkers and targeted therapeutic strategies for endometriosis.

There is an emerging evidence for direct link between peritoneal immune alterations and the microbiota. There are reports of changes in immune cell types in the peritoneum, such as neutrophils, macrophages, and NK cells, which are associated with changes in gut and genital microbiome compositions. This indicates the potential role of microbiota in regulating immune surveillance and tolerance, which could be favoring ectopic tissue implantation. Therapeutic interventions in microbiota composition, such as probiotics, antibiotics, and micro-ecological therapy have been reported to reduce serum level of IL-6/TNF, LPS and improve clinical outcomes in a clinical trial (25).

## 4 Antibiotic exposure and microbiota perturbation

Accumulating evidence indicates that antibiotics-mediated disruption of the microbiota plays a key role in modulating endometriosis associated inflammation and lesion development. Experiments conducted on animal model have proved that the alteration in the gut microbiome due to antibiotic use leads to the development of lesions and also affects the inflammatory responses and signaling in patients. In such cases, depletion of commensal bacteria through the administration of broad-spectrum antibiotics has been shown to reduce lesion size and decrease the production of pro- inflammatory cytokines in murine models, and this highlights the contribution of microbiota-driven immune activation in disease progression (26).

Bacterial exposure to broad-spectrum antibiotics significantly affects the microbiota diversity and balance, causing decreased microbial richness as well as imbalance within the gut microbial environment. This effect can lead to a change in systemic immune tone due to changes in microbial metabolites and modulatory signaling of immune system, causing alteration in macrophages, T-cell differentiation and cytokine secretion, all of which will have positive or negative impact on inflammation, depending on the timing of the dysbiosis and extent of damage (27). Antibiotic therapy as mentioned above causes an increased risk of chronic dysbiosis, which in turn might affect essential functions of the host-microbiota axis such as the activity of estrobolome. Microbial beta-glucuronidase enzyme activity can significantly influence the level of circulating estrogen hormone and contribute to maintaining and aggravating endometriosis lesions. Furthermore, dysbiosis is related to chronic low grade inflammation and enhanced intestinal permeability. With respect to translational aspects of manipulating microbiota through the use of antibiotics, there are numerous ethical and methodological issues. Although antibiotics provide excellent tools for uncovering the mechanisms underlying microbiota-immunity cross-talk, the clinical relevance of these studies is hampered to human beings as well as the possibility of having off-target, systemic effects due to antibiotics. Uncontrolled and excessive use of antibiotics can also promote resistance and disrupt the microbiota ecology, making it essential to explore other possibilities (12).

The antibiotic usage drastically impacts the composition of the gut microbiome and thus facilitates to the development of endometriosis by modulating immune regulation and inflammation due to changes in hormonal balance. Disruption in the functioning of the microbial community leads to low level of beneficial metabolites and alteration in estrogen metabolism, affecting endocrine and immune pathways implicated in the initiation of endometriosis (12, 27, 28). Indeed, experimental research supports the hypothesis, showing that the alterations in the functioning of the administration of antibiotics result in reduced lesion growth and inflammation in animals with the disease (26). Given the significant disruptions to the microbiota caused by antibiotic exposure, increasing attention is being directed toward the use of probiotics, prebiotics, and other microbiota-targeted strategies to re-establish ecological balance and regulate host physiological responses.

Emerging preclinical evidence provides strong support for a causal involvement of gut microbiota in the development and progression of endometriosis through antibiotic-induced modulation of the intestinal microbial community. There are several studies have shown that depletion of gut microorganisms using broad-spectrum antibiotics markedly reduces endometriotic lesion volume, suppresses cellular proliferation, and attenuates the production of pro-inflammatory cytokines. From the antibiotics investigated, metronidazole consistently exhibited a pronounced inhibitory effect on lesion development, whereas neomycin produced little or no therapeutic benefit, proposing that anaerobic bacterial populations may have a particularly important role in the pathophysiology of endometriosis (26).Further mechanistic evidence has been provided by fecal microbiota transplantation (FMT) experiments, in which transplantation of fecal microbiota from endometriosis-bearing mice restored lesion growth and inflammatory cytokine production in antibiotic-treated recipients. These findings indicate the change in the gut microbiome actively contribute to the disease progression rather than simply reflecting secondary changes associated with endometriosis (29).

Nevertheless, the interpretation of these findings should be approached with caution due to considerable methodological heterogeneity and the predominance of animal-based evidence. Experimental study might differ with respect to antibiotic selection, treatment duration, dosage, route of administration, animal models, and outcome assessment, thereby limiting direct comparison across studies. In contrast, human evidence is currently derived mainly from observational investigations which often lack adequate control groups, randomization, and long-term follow-up. Sometimes, antibiotics influence several host physiological processes beyond microbial depletion including alteration in estrogen metabolism through modulation of beta-glucuronidase-producing bacterial populations, making it challenging to differentiate microbiota-dependent effects from broader metabolic consequences such as disruption of beneficial commensal microbes, impaired microbial resilience. Consequently, although antibiotic-induced microbial perturbation provides compelling proof-of-concept supporting the impact of gut microbiota in endometriosis pathogenesis. Current evidence is insufficient to recommend antibiotics as a definitive therapeutic approach. Upcoming researchers should focus on adequately powered randomised controlled trials employing standardised antibiotic protocols, longitudinal microbiome analyses multiomic approaches and clinical validation using targeted manipulation of gut microbiota can safely and effectively reduce inflammation and improve clinical outcomes in women with endometriosis.

## 5 Probiotics, prebiotics, and microbiota modulation

Upcoming evidence highlights the therapeutic potential of prebiotics, probiotics, and synbiotics, and in modulating microbiota-driven immune and inflammatory pathways implicated in endometriosis. Numerous experimental and clinical investigation indicates that the supplementation of beneficial microorganisms, especially those belonging to the genus *Lactobacillus* and *Bifidobacterium* possesses the ability to inhibit inflammatory reactions and affect the course of condition. Probiotic treatment in mice with endometriosis has proven effective in reducing lesion size, inhibiting the production of pro-inflammatory cytokines, and restoring the immune balance (26). On a mechanistic context, probiotic and prebiotic treatment act via multiple mechanisms. This includes increased population of SCFAs like butyrate and propionate that influence immune signaling and epithelial functions, inhibiting pro-inflammatory cytokines (IL-6, TNF-Alpha, IL-17) and increasing the stability of tight junction proteins and thus strengthening the intestinal barrier. Furthermore, probiotic bacteria affect macrophages polarization and T-cell development to promote immune homeostasis and minimise chronic inflammation (18). Comparisons of results obtained from animals and humans have both similarities and differences. In animal studies, a clear trend has been observed that probiotic supplementation has a suppressive effect on inflammation and lesion occurrence (30). However, clinical studies show inconsistent data due to different strains, doses and duration of treatment. However, emerging research indicates that probiotics have some beneficial effects for endometriosis patients in terms of reducing their symptoms. There are several limitations associated with the use of probiotics in endometriosis patients. Strain specificity and individual variability of the gut microbiome are among the main difficulties. The exact dose of probiotics, timing of the procedure, and even the form of bacteria are unknown, making it difficult to implement therapy (14).

Strategies targeting microbiota, including prebiotics, probiotics, diet changes, and FMT, have been suggested as potential treatment methods to control endometriosis pain. As discussed above, the function of the gut microbiota includes the regulation of the processes responsible for immunity, inflammation and the gut-brain axis and the perception of pain and central sensitazation (31).As illustrated in **Figure 4**, gut dysbiosis functions as a core motivation of endometriosis by promoting elevated chronic inflammation, increased intestinal permeability, immune dysregulation and estrogen recirculation. Moreover, dysbiosis was found to affect the process of endometriosis development due to its ability to cause immune dysregulation, inflammatory signaling and abnormal metabolism of estrogens through the estrobolome (32). Supplementation with probiotics was shown to provide benefits in terms of decreasing the growth rate lesions, inflammation and pain intensity. Other methods, such as dietary modification (including low-FODMAP dieting and the consumption of polyunsaturated fatty acids) and prebiotics can lead to changes in the composition of microbiota, favoring the growth of bacteria, producing short chain fatty acids and maintaining the immune balance. FMT has been proposed as a potential microbiome-targeted strategy for modulating visceral pain based on evidence froom other gastrointestinal conditions; however its efficacy and safety in endometriosis have not been established and no clinical trials in women with endoemtriois have been reported (31).Further support for the benefits of microbiome modification comes from studies on probiotics effect and dietary supplements such as omega-3-fatty acids, N-acetyl cysteine and curcumin. Their anti-inflammatory and anti-oxidative properties might result in decreased pain and reduced lesion size (33).

**Figure 4:**
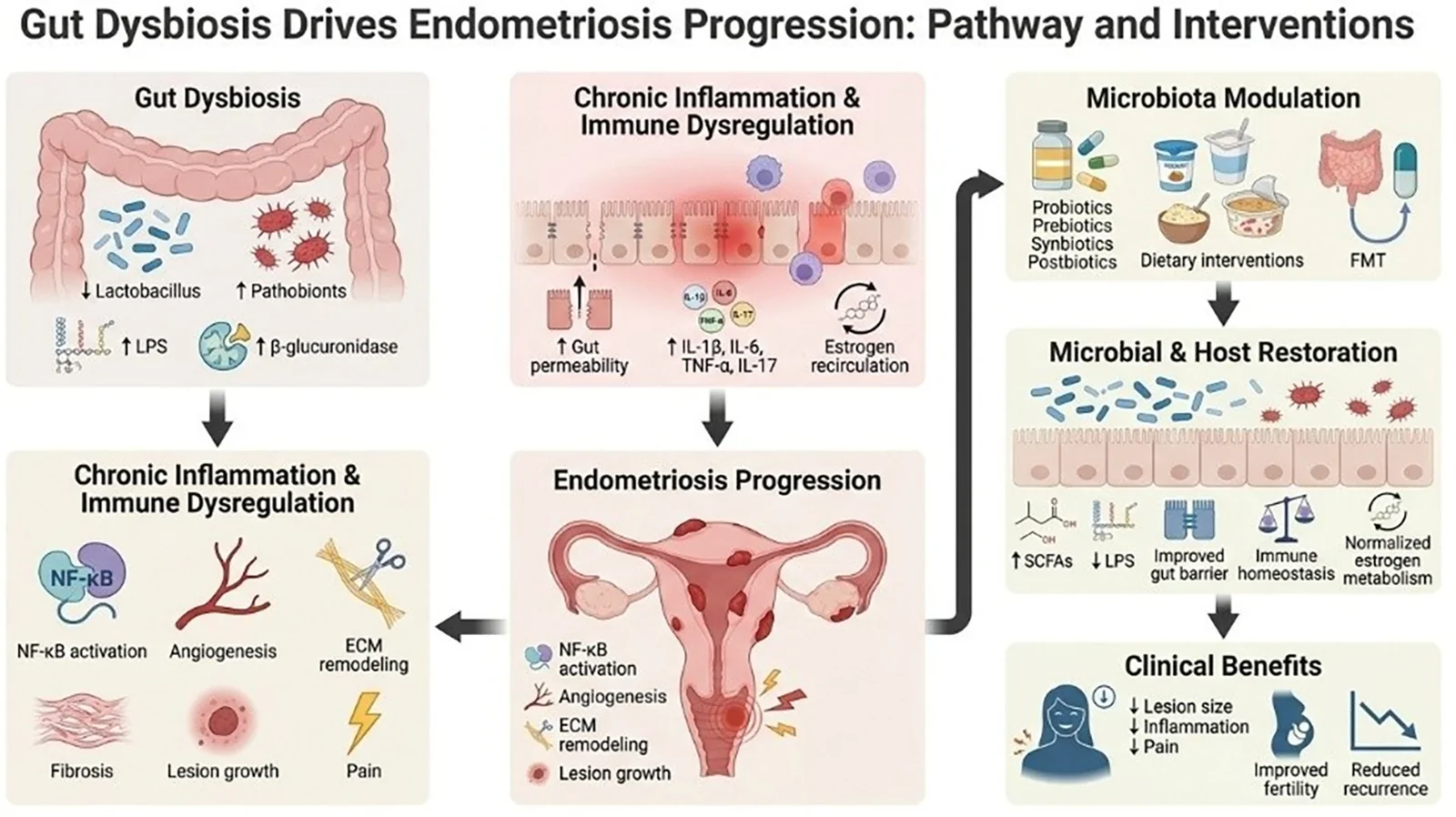
Gut-dysbiosis-endometriosis axis and microbiota-based therapeutic interventions. This figure explains the gut-dysbiosis contributes to chronic inflammation, immune dysregulation, altered estrogen metabolism, and disease progression. It also showing that’s prebiotics, probiotics, synbiotics and postbiotics, which aim to restore microbial homeostasis and improve clinical results in endometriosis.

Recent developments point towards the microbiome-based approaches are promising therapeutic non-invasive approach against endometriosis through regulation of microbial balance, immunity, and hormones. Gut dysbiosis has been linked to inflammation, immune system dysfunction, and alterations in estrogen metabolic processes, thus causing disease progression (34). The breakdown of the mucosal immune system structures like GALT and Peyer’s patches makes immune dysregulation worse and leads to heightened inflammatory processes. Modulation techniques, including prebiotics, probiotics, and fecal microbiota transplantations (FMT) have shown their ability to target these processes through boosting the beneficial bacteria, especially *Lactobacillus* strains, and decreasing pro-inflammatory bacteria. Such methods not only influence the immunity, but they also control the gut microbiota-estrogen pathway and metabolite production, like short-chain fatty acids (SCFAs) necessary for immune regulation (35). Moreover, the development of microbiome engineering methods, including diet-induced modifications, probiotic engineering, and microbiome transplantation, has the potential to affect not only gut but also vaginal microflora and consequently reproductive function (36). Although these approaches show great promise, their clinical application is still limited due to several obstacles, like lack of safety, standardization, diverse microbial profile, and individual variations. Finally, the search for the proper microbiome biomarkers and incorporation of multi-omic approaches, such as metabolomics, will play an important role in the emergence of personalised medicine. In conclusion, although microbiota-based therapeutics hold great potential, more research is necessary to prove their effectiveness and safety in practice.

Moreover, emerging studies continue to support the importance of microbiome modulation as an efficient therapeutic approach to improve reproduction and control endometriosis via modulation of the gut-reproductive axis. Crosstalk between the gut and reproductive tract bacteria are facilitated by bacterial metabolites such as short-chain fatty acids (SCFAs), bile acids, and indoles, controlling the immune system, inflammation, and hormones (37). Both gut and reproductive tract dysbiosis impairs immune surveillance, pro-inflammatory cytokine level increase, and results in angiogenesis and the progression of lesions in endometriosis (18). Probiotic and prebiotic treatment has shown to be useful for achieving microbial balance by promoting dominance of lactobacilli critical for sustaining the health of the reproductive tract and resisting infection. Clinical data also indicate that use of multi-strain probiotics can be beneficial to relieve pain and minimize the need for NSAIDs, yet their effect on inflammatory mediators and quality of life remains unclear (38). Finally, advanced microbiota manipulation approaches, including post-biotics and fecal microbiota transplantation (FMT) techniques, show great promise for dysbiosis correction and reproductive success but require careful assessment of safety, ethics, and regulations. Notwithstanding these positive developments, there are many challenges associated with methodological diversity, absence of standardized protocols, and difficulties in proving causality. In this regard, multi-omics analysis, large randomized controlled trials, and individualized strategies should be prioritized in future research.

These evidence indicates probiotics may offer an innovative therapeutic approach for the gut microbiota using probiotics may offer a novel therapeutic approach for the management of endometriosis by reducing inflammation and limiting disease progression. Experimental studies have consistently reported favorable outcomes following probiotic supplementation. Especially, *Lactobacillus gasseri* OLL2809 has been shown to significantly decrease the size of ectopic endometrial lesions. In certain experimental models, mainly *Saccharomyces boulardii*, either alone or combined with *Lactobacillus acidophilus*, has been associated with reductions in lesion volume, and attenuation of pain related behaviors. Although clinical evidence remained limited, preliminary studies suggest that *L.gasseri* OLL2809 may improve dysmenorrhea and menstrual pain in women with endometriosis, while convincing proofs demonstrating its ability disease progression is still lacking. Despite these encouraging findings, the available evidence is limited by considerable methodological variability. Differences in probiotic strains, dosages, study design and outcome measures make comparisons difficult. Most of the evidences are coming from animal, and the clinical trials are few, includes small sample sizes, and often rely on subjective symptom assessments. Majorly probiotic effects are strain specific and only few studies evaluated probiotic viability, colonization, or long terms changes in gut-microbiota. Evidence for prebiotics and synbiotics is also rare, despite their potential to promote beneficial bacteria and increase anti-inflammatory short chain fatty acids (SCFA) production. These studies are essential to determine whether microbiota-targeted therapies can safely and effectively reduce inflammation and slow endometriosis progression.

Eventhough probiotics have shown promising therapeutic potential in experimental models of endometriosis, their translation into clinical practice is limited. Potential mechanisms by which probiotics may benefit endometriosis include re-establishing microbial balance within the gut and reproductive tract, strengthening intestinal barrier function, and regulating both local and systemic immune responses (39). Clinical studies have shown that probiotic supplementation, particularly formulations containing *L. gasseri. L. rhamnosus, L.acidphilus, and L.reuteri* may alleviate dysmenorrhea, chronic pelvic pain, and improve quality of life while attenuating inflammatory responses. However, their effects on lesion regression, disease recurrence, and infertility remains inconclusive due to limited sample sizes, heterogeneity in probiotic strains and formulations, and methodological across clinical studies (40). Accordingly, current international clinical guidelines do not recommend probiotics as a standalone treatment for endometriosis; however, they are increasingly regarded as a promising adjunct to established therapies, surgery, dietary interventions and lifestyle modification. Beyond therapeutic modulation, microbiome research has opened new opportunities for non-invasive diagnosis. Reproducible microbial signatures like including depletion of *lactobacillus*, enrichment of *E. coli*, increased beta-glucuronidase activity and metabolite alterations have emerged as promising diagnostic biomarkers for disease detection, staging, and prognosis although none have yet achieved sufficient validation.

Considering the future, new approaches using microbiome, such as post-biotics, synthetic probiotics, and customized microbiota therapies, could be potential areas to explore in future investigations (41). With the advent of new technologies and precision medicine, it is anticipated that there will be an easier and more feasible way of developing effective treatments through microbial treatment methods for endometriosis and chronic inflammatory conditions (18). By elucidating the effects of prebiotics and probiotics on host-microbiota interactions requires in depth molecular level investigation which can be pave the way through the host gene expression profiling and the incorporation of multi-omics approaches. Overall, the available evidence supports the consideration of prebiotic and probiotic supplementation as biologically plausible and potentially safe adjunctive approaches, rather than as independent treatments for endometriosis. Preclinical studies and preliminary clinical investigations, including a randomised, double blind, placebo-controlled cross over trial of oral probiotics (15) and strain-specific evidence for *Lactobacillus gasseri* OLL2809 and *L.reuteri* RC-14 presented in **Table 2**, have reported improvements in dysmenorrhea and pelvic pain with generally favorable safety profiles. These findings suggest that such intervention may have value as complementary approaches alongside established hormonal and surgical therapies, rather as substitutes for standard treatment. Nevertheless, the interpretation of these findings is limited by small study populations, short treatment periods, variation between strains and intervention protocols, preventing the formulation of evidence based recommendations regarding particular strain, dosage, treatment duration. Future research should therefore prioritise adequately powered, well designed randomised controlled trials using clearly characterised and standardised strains, with clinically meaningful outcomes including lesion progression or recurrence, pain quality of life, and fertility. Such evidence will be essential before prebiotic or probiotic interventions can be appropriately incorporated into clinical guidelines for endometriosis.

**Table 2:** Effects of probiotic strains on endometriosis and related inflammatory conditions in various study models.

A. Human studies						
Sl.no.	Probiotic strain		Disease model	Administration route	Mechanism/key outcome relevant to endometriosis	Reference
1	*Lactobacillus gasseri* OLL2809		Endometriosis (women with dysmenorrhea)	Oral	NK-cell activation, IL-12 induction, Immune modulation.Significantly reduced dysmenorrhea and pelvic pain symptoms	(40)
2	*Lactobacillus reuteri RC-14*		Endometriosis associated dysbiosis	Oral/ Vaginal	Restoration of *lactobacillus* dominant microbiota in vaginal and gut microbial balance and reduction in inflammatory dysbiosis associated with endometriosis	(51)
3	Multi-strain *lactobacillus/bifidobacterium* formulations		Endometriosis associated pain	Oral	Improvement in gut microbiota composition and reduction of inflammatory markers associated with disease progression	(15)
B. Animal studies						
1	*Lactobacillus gasseri* OLL2809	Murine endometriosis model		Oral	Activation of NK cells and increased IL-2 signaling. Suppressed development of ectopic endometrial lesions	(40)
2	*Lactobacillus gasseri* OLL2809	Rat endometriosis model		Oral	Immune modulation and anti-inflammatory effects	(67)
3	*Lactobacillus rhamnosus* GG	Experimental intestinal inflammation		Oral	Gut barrier restoration and cytokine suppression helps in reduced inflammatory cytokines and improved immune homeostasis	(68)
4	*Bifidobacterium longum*	Inflammatory disorder		Oral	Treg activation and immune regulation	(69)
5	*Bifidobacterium spp*	Endometriosis associated dysbiosis		Oral	SCFA production and regulation of gut-immune axis and Reduced inflammation associated with lesion progression	(51)
6	*Saccharomyces boulardii* CNCM I-745	Gastrointestinal inflammatory disorders		Oral	Modulation of intestinal immunity and cytokine signaling	(70)

This table summarizes investigated in clinical and experimental models of endometriosis, including their administration routes, proposed mechanisms, and therapeutic effects on microbial homeostasis, immune regulation, inflammation and lesion progression.

## 6 Host gene expression and multi-omics integration

Transcriptomic technologies have significantly advanced the understanding of host gene regulation in reproductive and gastrointestinal tissues. Early studies using microarray and bulk RNA sequencing enabled the identification of differentially expressed genes (DEGs) associated with inflammation, angiogenesis, and cellular remodeling in endometrial disorders and intestinal diseases. Recent studies using single-cell RNA sequencing (scRNA-seq) technology have led to remarkable discoveries in defining transcriptional signature of each cell type. For example, large-scale scRNA-seq analysis of endometriosis tissues has led to the identification of epithelial, stromal, and immune cells with altered inflammatory and complement signaling in tissue sites (42). These approaches enable the construction cellular atlases that capture heterogeneity across diseased and healthy tissues. Additionally, emerging spatial transcriptomic technologies further enhance transcriptomic studies by preserving the spatial context of gene expression within tissues, allowing researchers to map transcriptional activity in specific microenvironments such as the endometrium or intestinal mucosa.

The microbiome plays a crucial role in regulating host gene expression and immune response modulation. Microbiota alterations leads to host transcriptional changes related to inflammation, metabolism, and immunomodulation. Studies conducted to investigate recurrent implantation failure revealed a connection between changes in endometrial microbiota and disruption of host gene expression regulation responsible for immunity adaptations and vascularization, which are crucial processes in the embryo implantation process (43).Furthermore, research concerning interactions between the microbiome and hosts in intestinal tissues indicated that microbial metabolites and signals could regulate host gene expression related to immune reaction and metabolism. These results indicating that microbiota dysbiosis can influence disease development through immune response and inflammation alterations. An increased interest is seen toward multi-omics approaches that involve analysis of multiple Omic layers, including microbial sequencing data and host transcriptomic and metabolomics analyses. Using such techniques it is possible to find connections between microbial populations and host gene expression changes. Metagenomics analysis and transcriptomics can help scientists to establish connections between microbial populations and host metabolic and immune pathways. Multi-omics approach enables a better understanding of microbial population impact on host health and diseases.

Advanced methods of computation are key when it comes to interpreting large-scale host–microbiome datasets. For example, weighted gene co-expression network analysis is one such method that provides researchers with an approach to detect modules of genes that will correlate to the microbial composition of an individual and any disease phenotype they may have. Networks that are driven by their genes will allow researchers to think of how host gene expression correlates with microbial functions; by doing this, it will provide insight into the molecular mechanisms that are used for host-microbe interactions (44). In addition, integrative methods such as multi-omics correlation analyses and machine learning network inference are becoming popular for research to explore complex relationships between microbial taxa, the transcriptomic profile of the host, and the metabolomic signatures of the host. As a result of these studies, integrating transcriptomic co-expression networks for dual transcriptomic studies provide the identification of modules comprised of both host and microbial genes, leading to increased insight into spatial heterogeneity and the nature of regulatory interactions in the intestinal environment (45). Together, these computational strategies provide a systems level understanding of host–microbiome dynamics and will ultimately help to identify possible biomarkers and therapeutic targets. The major omics platforms, analytical techniques, primary outputs, and their relevance to understanding host–microbiota interactions in endometriosis are summarized in **Table 3**.

**Table 3:** Integrated multi-omics approaches for analysing host gene expression and host–microbiota crosstalk in endometriosis.

Sl.no.	Omics layer	Techniques	Core biological insight	Primary outputs	Significance in endometriosis	References
1	Genomics	Whole genome sequencing(WGS),Genome wide association studies(GWAS)	Finds out the genetic variation linked to disease susceptibility	Single nucleotide polymorphisms(SNPs), Genomic mutations	Provide insight into host genetic factors that influence immune regulation and microbial composition	(49)
2	Transcriptomics	RNA Sequencing (bulk and single cell), microarrays and spatial transcriptomics	Evaluate gene expression dynamic across tissues and cell populations	Differentially expressed genes(DEGs), signaling pathways	Highlight the altered inflammatory and immune-related gene expression in endometriotic tissues	(47)
3	Epigenomics	DNA Methylation profiling, ChIP-seq, ATAC-seq	Explores regulatory mechanisms independent of DNA sequence changes	Epigenetic marks, chromatin accessibility patterns	Explains how environmental microbial factors modulate gene expression through epigenetic modifications	(57)
4	Proteomics	Liquid chromatography-mass spectrometry(LC-MS/MS).2D-Gel electrophoresis	Assess protein abundance and post-translational modifications	Protein expression profiles, modified proteins	Identifies key inflammatory mediators and signaling proteins involved in disease progression	(71)
5	Metabolomics	LC-MS,GC-MS,NMR	Characterizes metabolic alterations within biological systems	Metabolite signatures, pathway fluxes	Links the microbial-derived metabolites with host physiological responses	(26)
6	Metagenomics	16S rRNA gene sequencing, whole metagenome shotgun sequencing	Profiles microbial community composition and functional potential	Taxonomic distribution, microbial gene content	Reveals microbial dysbiosis in gut and reproductive tract associate with endometriosis	(72)
7	Metatransciptomics	Microbial RNA sequencing	Examine actively expressed genes	Functional gene expression profiles	Provides insight into real time microbial activity influencing host pathways	(42)
8	Multi-omics integration	Machine learning approaches,MOFA, Cytoscape	Integrates multi layer datasets for system level analysis	Interaction networks, predictive biomarkers	Enables the identification of complex host-microbiota interaction network and therapeutic targets	(73)

This table is an overview of multi-omics approaches to investigate the molecular and microbial mechanisms underlying endometriosis. The table summarizes major omics platforms, associated analytical techniques, key biological insights, primary outputs and their relevance to understanding the host-microbiota interactions, immune dysregulation, and molecular mechanisms involved in endometriosis.

Here the **Figure 5**, provides an integrated overview of molecular and cellular mechanisms undergoing endometriosis which highlights the interplay between molecular dysregulation, and immune microenvironment, and single-cell transcriptomic alterations. Transcriptomic studies consistently demonstrate increased expression of key inflammatory mediators, including NF kB1, RELA, TNF, IL1B, IL6, IL8, IL17 together with angiogenic factors such as VEGFA, HIF1A, and PDGF, and matrix remodeling enzymes (MMP2, MMP3, and MMP9). It clearly shows that reduced expression of ESR1, PGR, and HOXA10 reflects progesterone resistance, disrupted estrogen signaling, and diminished endometrial receptivity. System biology approaches, including protein- protein interaction (PPI) network analyses, Differential expression gene (DEG) analysis, and weighted gene co-expression gene analysis (WGCNA) have identified several hub genes including TAGLN, GATA6, CDH3, CLU, COL8A1, ATF3, PROK1, CXCL13, JUNB, DKK1, KLF4 which regulate immune response, ECM modeling, and steroid hormone signaling. Immune transcriptomic analysis reveals the enrichment of CD163+M2 macrophages, regulatory T cells, dendritic cells, and IL-10 regulating immunosuppressive pathways escorted by diminished cytotoxic CD8+ T-cell and NK cell function creating a continuous immune tolerant microenvironment. The advancement of the single cell RNA sequencing (scRNA-seq) have uncovered the unrecognized cellular heterogeneity, including StAR-positive fibroblasts involved in local estrogen biosynthesis, complement –activated macrophages, and exhausted lymphocyte subsets earlier. Spatial transcriptomics has further highlighted the spatial complexity of endoemetric lesions by showing the epithelial overexpression of XBP1, VCAN, and CLDN7, THBS1-enriched pre-vascular regions, and fibroblast specific fibrotic niches driven by WNT5A signaling. Altogether, integrated multi-omics studies have identified several promising biomarkers CLDN7, XBP1, VCAN, and CH13L1 with excellent diagnostic performance (AUC>0.90). All the transcriptomic studies validated using qPCR, Immunohistochemistry, ELISA, immune-fluorescence and independent patient cohorts. For substantial progress in the biomarkers we need large multicentre validation and standardized multi-omics pipelines before they can be translated into routine clinical diagnosis.

**Figure 5:**
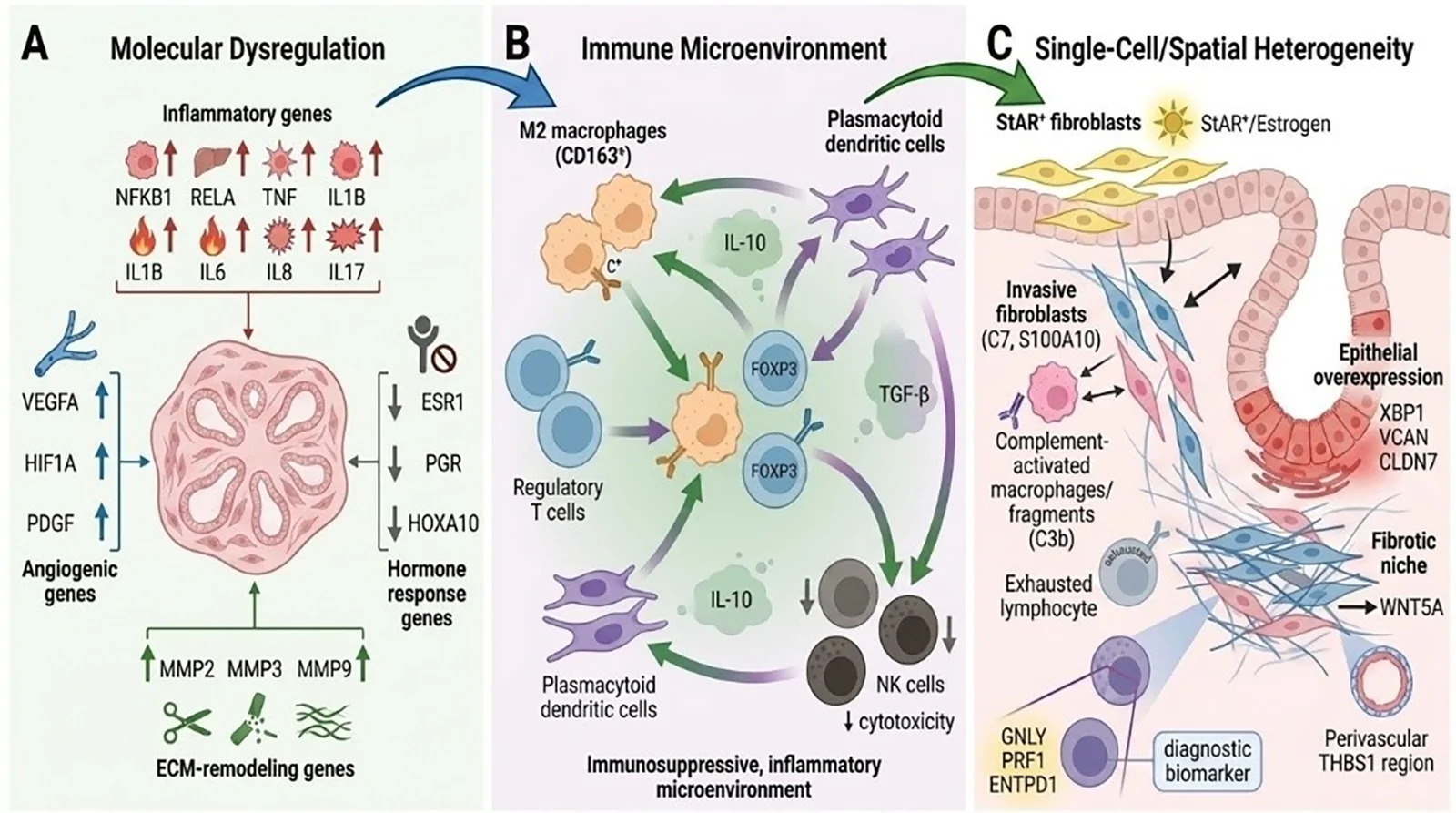
Integrated molecular, immune, and single-cell landscape of endometriosis. This figure integrates the molecular, immune, and single-cell landscapes of endometriosis, illustrating how dysregulated inflammatory signaling, extracellular matrix remodeling, angiogenesis, and hormonal imbalance interact with changes in the immune microenvironment to drive lesion establishment and progression.

The accumulating amount of scientific data indicates that the relationship between a host and its microbiome plays a significant role when examining reproductive health disorders, such as how these interactions can lead to altered gene expression and multi-omic integration. Numerous studies report that endometrial microbiota dysbiosis is associated with profound changes in host transcriptomic landscapes, leading to the potential disruption of key mechanisms needed for successful embryo implantation (43). In addition, the association between the microbiome and endometriosis is increasingly being recognised as a two-way relationship, with the imbalance of microbial populations contributing to chronic inflammation and immune dysfunction, while the effects of disease progression will further alter the composition of microbial community (46). The integration of metagenomics, epigenomics, and metabolomics has increased our knowledge of this interactions and allows for comprehensive studies of the host-microbe interface through simultaneous analysis across multiomic platforms (47). Frameworks that integrate the aforementioned omic analysis platforms with modeling tools, such as network analysis and regression-based methods, provide researchers with tools necessary for elucidating the complex, non-linear mechanisms involved in the pathogenesis of diseases.

Recent advances in transcriptomics, proteomics, metabolomics, and single-cell sequencing have substantially improved our concept in molecular mechanisms underlying endometriosis and their relationship with gut microbial dysbiosis. Multi-omics studies have adequately identified dysregulation of inflammatory, angiogenic and extracellular matrix remodeling pathways. It also includes the gut microbial metabolites directly influence host molecular pathways, with quinic acid promoting lesion growth and butyrate suppressing disease progression through anti-inflammatory signaling, highlighting the functional interplay between the gut microbiota and host gene regulation (48). Different sample types, sequencing platforms, and analytical pipelines together with small cohorts and limited matched microbiome host datasets, continue to reduce reproducibility and hinder cross study comparisons. Thereby implementing standardized workflows, larger longitudinal cohorts, and integrated microbiome host analyses with functional validation to clarify casual mechanisms and identify reliable biomarkers for endometriosis.

The field of multi-omics research has made significant advances and this reinforces the value of integrative models for studying host-microbiome relationships at an integrated systems level. Co-expression network analysis of dual transcriptomic datasets have revealed that microbial and host genes exist in highly connected modular structures, indicating coordinated regulation across biological systems (45). These type of studies have demonstrated that multiple microbial genes involved in metabolic pathways like polysaccharides degradation and amino acid synthesis can impact on the regulation of host immune genes, as well as the regulation of metabolic homeostasis. WGCNA-based methodologies have also identified strong associations between microbial communities and host genomics modules enriched in immune signaling pathways, as well as many important regulatory pathways (49). The field of Holo-omics has expanded our capacity to integrate data from a host and its microbiome in a single contextualized framework to better understand the biology of holobionts through clustering, correlations, and functional enrichment analyses (50). These findings support that interactions of the host with their microbiota are dynamic in nature, heterogeneous in spatial location, and dependent on key regulatory nodes of interaction, which ultimately provides critical insight for future biomarker development and precision medicine for endometriosis. Findings from host gene expression profiling and integrated multi-omics analyses not only clarify the molecular mechanisms underlying host-microbiota interactions but also emphasize the crucial influence of dietary factors in shaping the gut-endometriosis axis.

## 7 Dietary influences on gut–endometriosis axis

Diet plays a pivotal role in moulding the gut microbiota, which turn modulates key mechanisms underlying endometriosis pathogenesis (6, 51, 52). There is now considerable evidence to suggest that certain types of diets may induce changes in gut microbiota. Through these changes, dietary habits are capable of affecting estrogen metabolism, immune system reactions and inflammatory signaling. A western-type diet consisting of high concentrations of saturated fat and refined carbohydrates has been associated with gut dysbiosis, lower microbial diversity, and a greater variety of pro-inflammatory organisms. This dysbiotic condition results in greater β-glucurodinase activity within the estobolome, which results in the deconjugation and reabsorption of estrogen at levels that may lead to increased proliferation of ectopic endometrial tissue. By contrast, a fiber-rich, plant based diet would support the enrichment of beneficial microbes, such as those which produce anti-inflammatory short chain fatty acids (SCFAs), and play a key role in preserving intestinal barrier integrity.

Hormonal modulation along with dietary patterns also affect the gut’s barrier function, which influences immune activation and chronic inflammation associated with endometriosis. Eventually, an unhealthy diet can result in dysbiosis, which changes how tight junction protein work in the intestinal epithelial cells and can increase the permeability of the intestine, known as “leaky gut”. This allows microbial byproduct such as lipopolysaccharides(LPS) to enter the bloodstream through the intestinal and translocate into the bloodstream, resulting in the activation of toll-like receptor(TLR) pathways and downstream signaling through NF-kB (53). When TLRs are activated, cytokines like IL-6, TNF-alpha, and IL-1B are released, producing a chronic inflammatory environment that can enhance the development and disease progression of ectopic endometrium. In addition, certain foods like trans fats and foods with high glycemic index, will worsen oxidative stress responses and inflammatory signaling, whereas nutrients like vitamin D and E, Flavonoids, and omega-3-fatty acids will reduce inflammation will also decrease these responses by minimizing inflammatory responses through modulating the inflammatory process and cytokine release from immune cells (54). The effects of dietary components on gut microbial composition, associated mechanisms, and their potential influence on endometriosis progression are summarized in **Table 4**.

**Table 4:** Dietary influences on the gut–endometriosis axis.

Sl. no.	Dietary factor	Major components	Influence on microbiota	Mechanism	Effect on endometriosis	References
1	High fat diet	Saturated fats, Trans fats	Reduced microbial diversity with enrichment of proteobacteria	Elevated LPS levels trigger activation of NF-kB signaling pathway, leading to chronic inflammation	Facilitates lesion establishment and growth	(33)
2	Low-fiber diet	Refined carbs	Depletion of SCFA producing bacterial populations	Reduced butyrate synthesis compromises intestinal barrier integrity	Accelerate disease progression	(74)
3	High fiber diet	Fruits, vegetable, whole grains	enrichment of SCFA –producing Microbiota	SCFAs promote regulatory T-cell responses and exert anti-inflammatory effects	Associated with reduced disease severity	(74)
4	Omega -3 fatty acids	EPA,DHA	Enhancement of beneficial microbial populations	Supression of prostaglandin E2 Synthesis and inflammatory pathways	Contribute to alleviation of pain and inflamamtion	(75)
5	Red meat consumption	Processed/red meat	Increased abundance of bile-tolerant bacteria such as bacteroides	Elevated beta –glucuronidase activity promotes estrogen reactivation and circulation	Linked to higher risk of endometriosis	(56)
6	Dairy products	Milk, curd, yogurt	Modulatory effect on microbial composition	Calcium and vitamin D contribute immune regulation	May exert protective effects though evidence remains inconsistent	(76, 77)
7	Probiotics	*Lactobacillus*, *Bifidobacterium*	Restoration of microbial homeostasis	Reducing of pro-inflammatory cytokines and strengthening of gut barrier function	Improves symptoms and reduces inflammation	(78–80)
8	Prebiotics	Inulin, fructooligosaccharides(FOS)	Selective stimulaton of beneficial microbes	Increased SCFA production supports immune and gut health	Enhances gut-immune interactions	(81)
9	Phytoestrogens	Soy derived isoflavaones	Modulation of estrogen metabolizing microbiota	Competes with endogenous estrogen at receptor level	May inhibit lesion progression	(81)
10	Alcohol	Ethanol	Induces microbial imbalance	Promotes oxidative stress and inflammatory signaling	Associated with increased disease susceptibility	(82)
11	Gluten	Wheat proteins	Alters gut microbial composition	Triggers immune-mediated inflammatory responses	Symptom relief observed upon exclusion in specific individuals	(55)
12	Antioxidant-rich diet	Vitamin C,E, polyphenols	Enhances microbial diversity	Reduces oxidative stress and inflammatory burden	May slow disease progression	(80)

The table summarizes dietary components, their effects on gut microbial composition, underlying mechanisms, and their potential impact on endometriosis progression, highlighting the role of diet in modulating host-microbiota interactions, inflammation, estrogen metabolism, and disease outcomes.

Recent studies have shown that dietary changes can affect the gut microbiota and assist in reducing the symptoms of endometriosis and slowing the progression of the disease. Both the clinical and experimental studies diets with specific macronutrients such as low fermentable oligosacharrides, disaccharides, monosaccharides and polyols(FODMAPs), Mediterranean-type diets, and probiotic/prebiotic supplementation can restore the balance of microbiota and decrease inflammation in the body. For example, low FODMAP diets help by providing fewer fermentable substrates to pathogenic bacteria in gastrointestinal tract and thereby lessening gastrointestinal symptoms associated with endometriosis. Consumption of fruits, vegetables, green tea, and other foods that are rich in polyphenols has been shown to elevate the microbial diversity of the gut and boost the production of anti-inflammatory metabolites (54, 55). Researchers have also utilized multi-omics approaches that combine metagenomics, metabolomics, and transcriptomics to demonstrate that the microbial metabolites produced as a result of dietary intake, such as bile acid and short chain fatty acid(SCFA),play an important role I regulating the gene expression of host tissues and regulating immune pathways believed to contribute to endometriosis (56). These results shows that diet is an important adjustable factor contributing to the gut-endometriosis axis and may help lead to the discovery of custom dietary therapies to treat endometriosis, creating new and unique ways to manage the disease. Considering the substantial influence of dietary factors on the gut-endometriosis axis, the application of rigorous experimental and analytical approaches in critical to cover the underlying mechanisms and substantiate these associations.

## 8 Experimental and analytical methodologies

An integrated approach that combines advanced experimental techniques with advanced computational techniques is important to get better elucidate the complex interactions between human biology and microbes living in or on humans. Understanding both the physiological, microbiological and clinical manifestations of the microbiome related to endometriosis will provide a more complete understanding of the functional and biochemical properties of the microbiome and its impact on human health. By using modern DNA sequencing technology and employing metabolic techniques, researchers will gain multidimensional understanding of the behavior of host-microbiome interactions. Together these methodologies facilitate the identification of microbial dysbiosis, elucidation of mechanistic pathways, and validation of casual relationships, thereby contributing to improved diagnostics and therapeutic strategies.

Research finding of the gut microbiota-endometriosis axis is characterized by substantial methodological variability, contributing to inconsistent findings and limited reproducibility. Studies differ in sample types, sequencing technologies, pipelines, reference databases, and analytical methods while important factors such as diet, BMI, hormone therapy, menstrual cycle phase, and antibiotic exposure are often inadequately controlled. Although advances in metagenomics, transcriptomics, metabolomics, proteomics, and experimental animal models have improved our understanding of host-microbiome interactions and given evidences that links gut dysbiosis to endometriosis. Future research should emphasize harmonized study designs, standard samples and bioinformatics workflows, comprehensive metadata reporting and large multicentre multi-omics studies enhance reproducibility and establish clinically important microbial biomarkers and therapeutic targets.

### 8.1 Microbiome profiling

#### 8.1.1 16S rRNA sequencing

As a widely adopted microbiome profiling technique, 16S rRNA gene sequencing is extensively used to characterize the taxonomic composition and diversity of microbial communities across a wide range of environments (**Figure 6**). This method is targets conserved regions within the bacterial 16S rRNA gene to classify microorganisms present in complex biological samples. Owing to its cost-effectiveness, relatively simple library preparation, and scalability of large cohort studies, 16S rRNA sequencing has become a standard approach for investigating microbial communities. It mainly valuable for comparing microbial diversity and community composition across different niches, including endometrium, gut, and peritoneal activity. Using this technique, several studies have identified microbial imbalances in endometriosis, including alterations in the abundance of key bacterial taxa such as *Lactobacillus* and *bacteroides* (57). However, the method is limited in its ability to achieve species-level resolution and does not provide detailed information into microbial functional capabilities, prompting the adoption of more comprehensive approaches like shotgun metagenomics.

**Figure 6:**
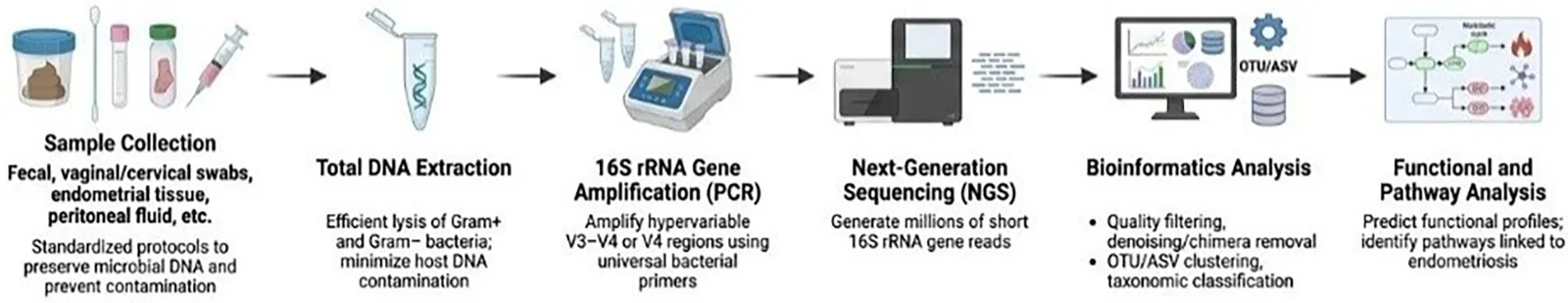
Workflow of 16S rRNA gene sequencing for microbiome profiling in endometriosis. This figure illustrates the workflow of 16S rRNA gene sequencing for microbiome profiling in endometriosis, incorporating sample collection, DNA extraction, PCR amplification, next generation sequencing, and bioinformatics analysis.

#### 8.1.2 Shotgun metagenomics

Unlike targeted sequencing approaches, shotgun metagenomics sequencing is a comprehensive experimental technique that analyses the entire microbial DNA content within a sample, providing both taxonomic and functional information. By capturing all microbial DNA, this approach enables simultaneous characterization of microbial taxonomic composition and functional potential (52). This technique also enables genome reconstruction and identification of key functional processes such as lipopolysaccharide production, amino acid biosynthesis, and bile acid transformation offering a more comprehensive understanding of microbiome-driven disease mechanisms (**Figure 7**). Despite these advantages, to elucidating microbiota-related mechanisms in endometriosis this method presently confined to experimental research settings and has not achieved widespread clinical utility for diagnostic purposes.

**Figure 7:**
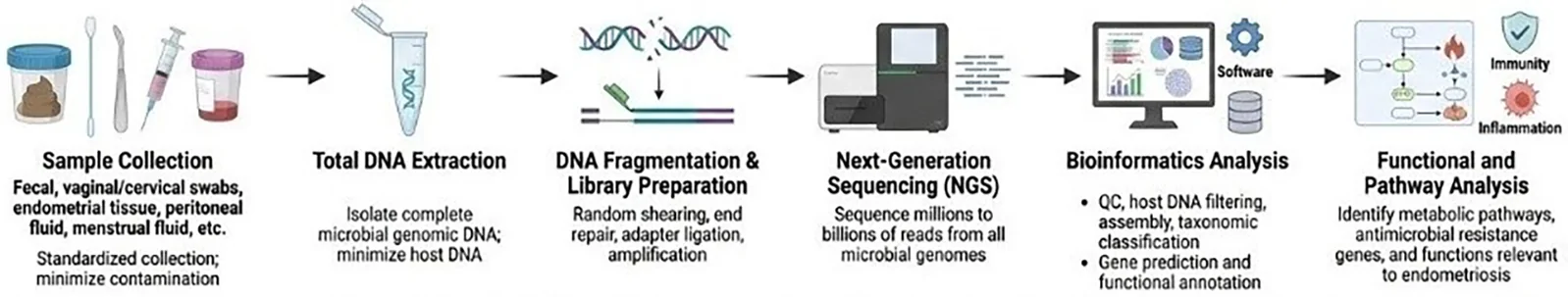
Schematic representation of metabolite extraction, profiling (LC-MS, GC-MS, NMR), and Data analysis pipeline. This figure presents a schematic workflow of metabolite extraction, metabolomics profiling using LC-MS, GC-MS, and NMR followed by data pre-processing, statistical analysis, and pathway interpretation.

#### 8.1.3 Metabolomics (LC-MS, GC-MS, NMR)

Metabolomics has emerged as a powerful experimental approach for investigating the functional consequences of host-microbiome interactions through the comprehensive analysis of small-molecule techniques such as liquid chromatography-mass spectrometry (LC-MS), Gas chromatography-mass spectrometry (GC-MS), and nuclear magnetic resonance (NMR) spectroscopy allow for detailed profiling of metabolites in biological samples. The **Figure 8** illustrates the comprehensive metabolomics workflow commonly employed to characterize metabolic alterations associated with endometriosis. Its primary value lies in generating hypotheses and identifying potential metabolic signatures that may guide future translational studies. Key compounds identified through these methods include short-chain fatty acids (SCFAs), bile acids and tryptophan derivatives, all of which play essential roles in immune regulation, maintenance of intestinal barrier integrity, and hormonal balance. For instance, SCFAs like butyrate and propionate exhibit anti-inflammatory properties, while disruptions in bile acids metabolism may influence estrogen signaling and inflammatory pathways associated with endometriosis (58). By linking microbial metabolic activity with host physiological responses, metabolomics provides a functional bridge between microbial community composition and disease- associated biological processes.

**Figure 8:**
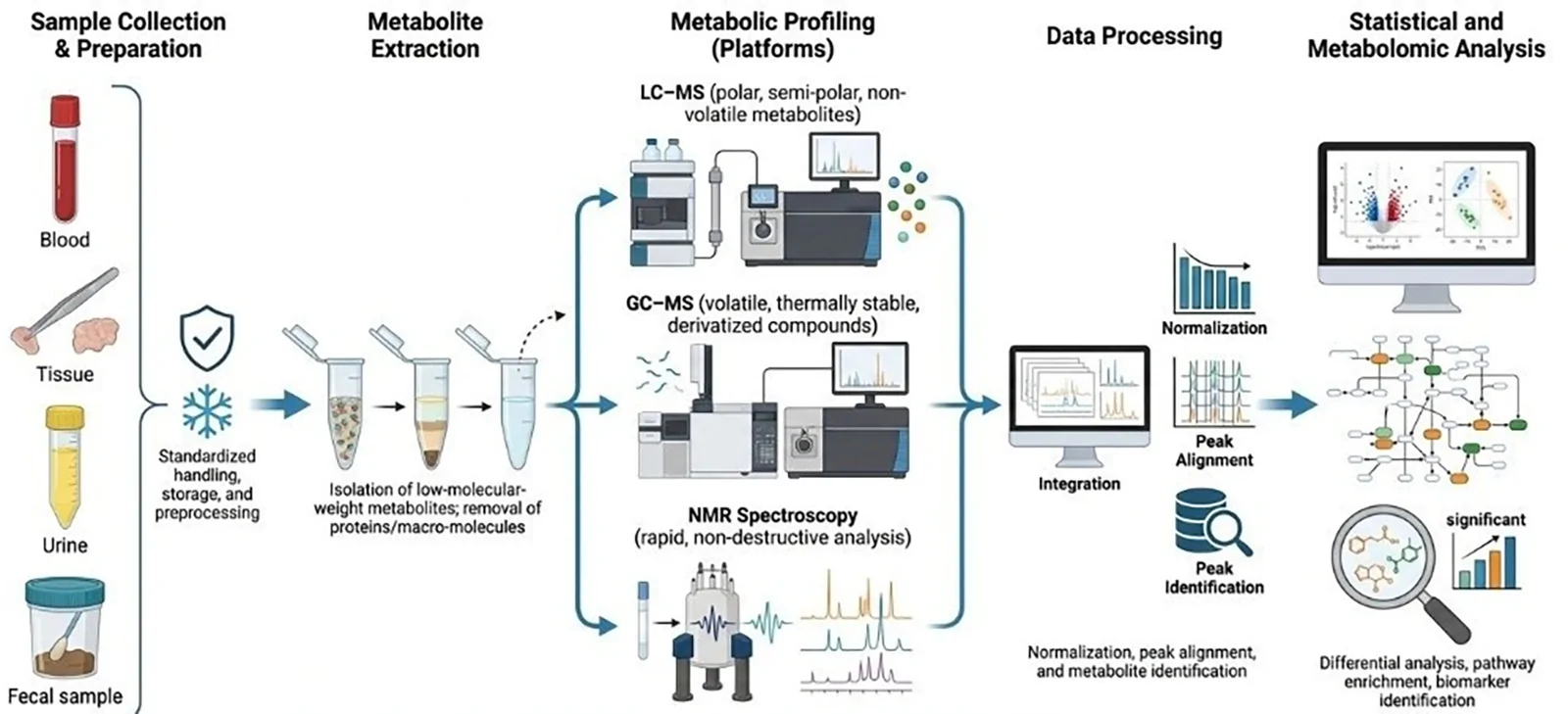
Schematic representation of metabolite extraction, profiling (LC-MS, GC-MS, NMR), and Data analysis pipeline. This figure explains the workflow of metabolomics analysis, including the sample collection, metabolite extraction, profiling by LC-MS, GC-MS, NMR followed by data pre-processing, statistical analysis, and pathway enrichment. This integrated pipeline enables comprehensive characterization of metabolic alteration, facilitating the identification of disease-associated biomarkers and metabolomics pathways involved in the pathogenesis of endometriosis.

#### 8.1.4 Fecal microbiota transplantation

Fecal microbiota transplantation is valuable experimental approach for investigating the causal relationship between microbial dysbiosis and disease. In preclinical studies, microbial communities obtained from donor fecal samples are transferred into germ-free or antibiotic-treated animal models to evaluate their effects on disease associated phenotypes. Studies using FMT have demonstrated that transplantation of microbiota from individuals with endometriosis can intensify inflammation and promote lesion development in recipient animals, indicating a direct role of the microbiome in disease pathogenesis (59). Despite these promising findings, FMT remains an experimental strategy in the context of endometriosis and has not been established as a routine therapeutic intervention. At present, FMT serves mainly as a research tool for understanding microbiome-host relationships and evaluating emerging microbiome-directed therapeutic approaches. As well as the FMT models serve as valuable platforms for testing therapeutic strategies, including probiotics and dietary interventions, aimed at restoring microbial equilibrium and mitigating disease severity.

## 9 Translational and therapeutic implications

### 9.1 Gut–endometriosis crosstalk and diagnostic biomarkers

A deeper understanding the bidirectional crosstalk between the gut microbiota and endometriosis pathology has created new opportunities for translational research, particularly in the discovery of diagnostic biomarkers and the development of precision medicine strategies. Accumulating evidence depicts that gut microbial dysbiosis contributes to immune dysregulation, chronic inflammation, and altered estrogen metabolism, all of which are central to the pathogenesis and progression of endometriosis (44). Specific microbial taxa, including *Blautia, Prevotella*, and other inflammation- associated with disease severity and inflammatory status in women with endometriosis (32). In addition, microbial metabolites produced by the gut microbiota may modulate the estrobolome, a group of bacterial genes involved in estrogen metabolism, thereby regulating systemic estrogen levels and potentially contributing to lesion growth and hormonal imbalance (18).Because of these microbial signatures can be detected in stool or reproductive tract samples, microbiome profiling has emerged as a promising non-invasive strategy for early disease detection and patient stratification. Advances in machine learning and multi-omics integration may further enable the discovery of microbiome-derived biomarkers that could reinforce personalized diagnostics and improve clinical decision-making (47).

### 9.2 Microbiome-targeted therapeutic strategies

Upcoming evidences indicates that modulation of the gut microbiota may offer a promising therapeutic strategy for management of endometriosis. A range of microbiome-based therapies such as probiotics, prebiotics, dietary interventions, and fecal microbiota transplantation (FMT) have been investigated for their potential to restore microbial balance and reduce inflammation (60). Probiotic supplementation, particularly strains of *Lactobacillus*, has shown promise in improving microbial composition and reducing inflammatory cytokine levels in experimental models and preliminary clinical studies (38). These beneficial microorganisms may strengthen intestinal barrier integrity, regulate immune responses, and mitigate the inflammatory microenvironment associated with endometriotic lesions (61). Similarly, dietary interventions, such as increasing dietary fiber intake and adopting anti-inflammatory dietary patterns, can promote the growth of beneficial gut microbes, enhance microbial homeostasis, and potentially slow disease progression (29). Fecal microbiota transplantation has also attracted considerable interest as an experimental microbiome based intervention. Preclinical studies showing that transferring dysbiotic microbiota from endometriosis models to recipients animals can alter disease severity, providing evidence for a causal role of gut microbiome in endometriosis pathogenesis. Although clinical application in endometriosis remains experimental, these findings highlight the potential of microbiome-targeted therapies as adjunct strategies for disease management.

### 9.3 Personalized medicine and precision microbiome interventions

The integration of microbiome data with host genomic, transcriptomic, and metabolomic profiles may facilitate the development of precision medicine approaches for endometriosis. Personalized microbiome interventions aim to tailor treatments based on an individual’s microbial composition and metabolic profile. For example, identifying specific microbial patterns associated with inflammation or estrogen metabolism could enable clinicians to design targeted interventions such as personalized probiotic formulations, dietary modifications, or microbiota restoration therapies (62). Emerging research suggests that microbiome-driven approaches may improve patient outcomes by addressing disease heterogeneity and tailoring treatment strategies to individual biological profiles (31). Such personalized strategies may also help identify patients who are most likely to benefit from microbiome-modulating therapies, improving therapeutic efficacy and reducing unnecessary treatments.

Artificial intelligence (AI) and machine learning offer powerful tools to incorporating these multi-omics datasets identify microbial and molecular signatures associated with disease susceptibility, stage, recurrence, and infertility. AI-driven models synthesizing the gut, cervical and endometrial microbiome profiles with clinical imaging and molecular biomarkers may facilitate the development of precise diagnostic and prognostic tools (47). Nascent technologies, including single-cell and spatial multi-omics, are expected to transform understanding of endometriosis by resolving chromatin accessibility, metabolite distribution, and cell-cell interactions within the lesion microenvironment. Integration of these datasets with microbiome-derived metabolites will provide a novel insight into immune dysregulation, while enabling the discovery of novel biomarkers beyond those identifies through bulk transcriptomic analyses (42). Another technological advances in long-read meta-genomic sequencing will again improves the microbial characterization by providing species and strain-level resolution, immune regulation, complete microbial genome assembly, and improved functional annotation of pathways involved in estrogen metabolism. These improvements will overcome many limitation of conventional 16S rRNA sequencing and more accurate characterization of microbial communities associated with endometriosis. Another promising avenue is development of digital twin model that integrate longitudinal clinical data, imaging,immune signatures and multiomic datasets to generate individualized computational model of disease. It can even predict the disease progression, risk, and therapeutic response while supporting personalized selection. Similarly, emerging microbiome- based therapies, including engineered probiotics and synthetic microbial consortia, hold promise for selectively modulating inflammatory signaling are currently confined to preclinical investigation. Ultimately, translating microbiome research into clinical practice will require large, multicentre longitudinal studies incorporating standard samples, protocols, validated sequencing, and independent external validation cohorts. In the future, integrating AI- assisted analytics with advanced multiomics technologies and microbiome based therapeutics will be essential for the validating microbial biomarkers and accelerating the development for early diagnosis, prognosis, and personalized treatment of endometriosis.

### 9.4 Ethical and clinical considerations in microbiome manipulation

Despite the promising potential of microbiome-based therapies, several ethical and clinical considerations must be addressed before their widespread clinical adoption. Manipulating the human microbiota raises concerns regarding long-term safety, donor selection, microbial transfer risks, and regulatory oversight, particularly in the context of procedures such as fecal microbiota transplantation (FMT). While FMT is already approved for treating recurrent *Clostridioides* difficile infection, its application in other diseases remains under investigation and requires rigorous clinical validation (63). Furthermore, the increasing use of microbiome profiling alongside genomic and multi-omics technologies necessitates stringent measures to protect patient privacy, ensure responsible data governance, and maintain ethical management of sensitive biological data. Additionally, microbiome-based diagnostics and treatments must ensure patient privacy and responsible data management, especially when genomic and multi-omics data are involved. Standardized protocols, longitudinal clinical trials, and ethical guidelines will therefore be essential to ensure the safe translation of microbiome research into clinical practice. Addressing these challenges will be crucial for realizing the full therapeutic potential of microbiome-targeted interventions in endometriosis and other chronic inflammatory diseases.

## 10 Discussion

The evidence synthesized in this review converges on a consistent, mechanistically plausible model of the gut-microbiota-endometriosis axis: gut dysbiosis, characterized reduced microbial diversity, depletion of *Lactobacillus*, and enrichment of pro-inflammatory taxa, compromises intestinal barrier integrity and permits low grade endotoxemia. Circulating LPS engages TLR4 on immune and epithelial cells, driving NF-KB- dependent production of TNF-α, IL-6, IL-1B, and IL-17, which in turn sustains macrophage recruitment, Treg/Th17 imbalance, and impaired NK-cell cytotoxicity within the peritoneal cavity. This inflammatory milleu does not act in isolation from endocrine signaling: microbial β-glucuronidase activity within the estrobolome deconjugates and reactivates estrogen metabolites, reinforcing local estradiol availability and thereby coupling microbial dysbiosis directly to the estrogen- dependent proliferation of ectopic lesions. Host transcriptomic and single-cell data reinforce this picture, showing that the same lesions marked by microbial and cytokine dysregulation also display reduced ESR1/PGR/HOXA10 expression, upregulated angiogenic and matrix-remodeling programs, and an immune- tolerant microenvironment enriched for M2 macrophages and regulatory T cells (18–20). Altogether, these findings support the view that endometriosis is not merely a local inflammatory lesion but a systemic condition shaped by continuous, bidirectional crosstalk between the intestinal microbiota, the immune system, and host regulation.

Casual evidence for this axis is strongest in animal models, where antibiotic- induced microbial depletion reduces lesion size and inflammatory cytokine output, and where fecal microbiota transplantation from endometriosis- bearing donors reproduces lesion growth and inflammation in recipients. These experiments demonstrate that the microbiota actively contributes to the disease progression rather than merely reflecting it. Probiotic supplementation, particularly with *Lactobacillus* and *Bifidobacterium* strains, produces converging benefits across both animal and early clinical studies, including reduced lesion volume, restored epithelial barrier function, and modest symptomatic improvement in dysmenorrhea and pelvic pain, and largely through SCFA-mediated anti-inflammatory signaling (26). Dietary modulation of the same pathways, through fiber intake, low FODMAP patterns, and anti-inflammatory nutrients, further supports diet as a modifiable determinant of the axis rather than a peripheral lifestyle factor.

Despite these findings, their translational potential remains limited. Much of the mechanistic evidence, particularly regarding antibiotic mediated microbial depletion and FMT, is based on animal models, and their applicability to the human pelvic microbiota and endometriotic lesion biology remains uncertain. Clinical studies are generally small, cross-sectional, and heterogeneous with respect to probiotic strains, dosages, and treatment durations, making meta-analytic synthesis difficult and limiting the ability to establish casual relationships. Furthermore, important confounding factors, including menstrual cycle phase, hormonal therapy, BMI, diet, and prior antibiotic exposure, are inconsistently addressed across studies. A detailed evaluation of the individual studies contributing to this evidence base emphasizes several important limitations. Human studies often involve relatively small sample sizes, which may reduce the statistical power for identifying robust microbiota-disease association and increase the likelihood of spurious taxonomic differences. Moreover, many case-control studies do not adequately account for important confounding factors that can independently influence the gut and reproductive-tract microbiota, including recent antibiotic exposure, hormonal contraceptive use, dietary patterns, and menstrual cycle phase at the time of sampling. Consequently it can be difficult to determine whether reported microbial alterations are specifically associated with endometriosis. The existing methodological heterogeneity also exists in microbiome profiling, with differences in 16s rRNA gene target regions, sequencing platforms, bioinformatics workflows, and taxonomic databases, limiting comparability between studies and complicating integrated analyses. Probiotic studies are additionally constrained by small cohorts, short intervention periods, and limited long-terms assessment of outcome such as lesion recurrence and fertility. Collectively these study level limitations contribute to the current uncertainty in the field and help why a reproducible, clinically validated microbiota signature or microbiota- based intervention for endometriosis has yet to be established. Although the multiomics investigations have identified promising microbial and transcriptomic biomarkers, including CLDN7, XBP1, VCAN, and CHI3L1, with promising diagnostic performance, these candidates have not yet been validated in large multicentre cohorts (16). Consequently, no microbiota- based biomarker signature or probiotic intervention has yet achieved sufficient evidence to support its incorporation into clinical guidelines. The discussion has been further strengthened by incorporating the implication of the PRISMA- based study selection and synthesis, thereby improving the transparency, rigor, and overall quality of the review.

These limitations chart the fields immediate priorities, progress will depend on adequately powered, randomized, placebo-controlled trials of standardized probiotic and dietary interventions; longitudinal, multiomics cohorts that integrate microbiome, transcriptomic, and metabolomics data with inconsistent sampling and sequencing protocols; and mechanistic human studies capable of testing causality rather than association. The emergence of AI-assisted multiomics integration, single cell and spatial transcriptomics and long-read metagenomic sequencing offers a realistic path toward this standardization, provided findings are validated across independent, multicentre cohorts before informing personalized probiotic or dietary regimens (43). Ethical and regulatory frameworks for microbiome-based interventions, particularly FMT, will also need to mature in parallel with scientific itself.

## 11 Conclusion

This review consolidates evidence across immunology, microbiology, transcriptomics, and nutrition to support the gut microbiota as an active, mechanistically integrated contributor to endometriosis pathogenesis rather than an incidental correlate of disease. By linking microbial dysbiosis to chronic inflammation, estrogen dysregulation, and host transcriptional reprogramming within a single conceptual framework, this work addresses a gap left by prior reviews that examined these domains in isolation. The clinical translation of this framework remains at an early stage; probiotic, dietary modification, and fecal microbiota transplantation show biological plausibility and preliminary benefit but are not yet supported by evidence sufficient for standalone clinical use, and microbiome-derived diagnostic biomarkers require further validation before informing patient care. Realizing the potential of the gut-microbiota-endometriosis axis for non-hormonal, personalized management will require standardized, adequately powered clinical trials and integrated multi-omics research, positioning microbiota-targeted strategies as a promising adjunct to, rather than replacement for, existing hormonal and surgical approaches to endometriosis.
